# Machine learning, molecular docking, and experimental validation identify shared molecular signatures between idiopathic pulmonary arterial hypertension and malignant pleural mesothelioma: a retrospective study

**DOI:** 10.3389/fgene.2026.1894027

**Published:** 2026-10-14

**Authors:** Neng Zhang, Chunyu Wang, Rui Wang, Xue Guo, Jing Yu, Jie Wang, Qin Luo, Yun Wu

**Affiliations:** 1 Department of General Medicine, The First Affiliated Hospital of Xinjiang Medical University, Urumqi, China; 2 Department of Emergency, Peking University People’s Hospital, Beijing, China; 3 College of Pharmacy, Xinjiang Medical University, Urumqi, China; 4 Department of Pharmacy, The First Affiliated Hospital of Xinjiang Medical University, Urumqi, China; 5 Department of Respiratory Neurology, The Second Affiliated Hospital of Xinjiang Medical University, Urumqi, China

**Keywords:** experimental validation, idiopathic pulmonary arterial hypertension, machine learning, malignant pleural mesothelioma, molecular docking

## Abstract

**Background:**

Idiopathic pulmonary arterial hypertension (IPAH) exhibits cancer-like features, yet its pathogenesis remains unclear. Given molecular similarities between IPAH and malignant pleural mesothelioma (MPM), this study aimed to uncover shared transcriptomic patterns.

**Methods:**

GEO datasets for IPAH and MPM were analyzed. Overlapping DEGs and WGCNA modules were identified. PPI network analysis combined with LASSO, random forest, and SVM-RFE was used to identify candidate biomarker genes. A classification model was constructed and evaluated using leave-one-out cross-validation and bootstrap optimism correction. Immune infiltration was evaluated by ssGSEA. Molecular docking and MD simulations assessed drug binding to candidate target proteins. qRT-PCR validated candidate biomarker gene expression.

**Results:**

Six concordantly upregulated and eleven concordantly downregulated DEGs were shared between IPAH and MPM. Upregulated genes were enriched in cell cycle regulation, while downregulated genes were enriched in neutrophil chemotaxis. WGCNA identified 58 overlapping genes. CXCR2, S100A9, and LCN2 were identified as candidate biomarker genes. A ROTS sensitivity analysis confirmed that all 1710 MPM DEGs were reproducible and directionally concordant at the matching FDR <0.05 threshold despite the imbalanced case–control ratio. The classification model achieved AUCs of 0.909 and 0.875 in training and external validation sets, with a LOOCV AUC of 0.879 and an optimism-corrected AUC of 0.896. Immune infiltration revealed increased Th17 cells but decreased neutrophils in IPAH. CXCR2 and S100A9 expression correlated with neutrophil infiltration; LCN2 expression correlated with metabolic reprogramming. Navarixin showed the strongest binding affinity to CXCR2 (−10.1 kcal/mol). MD simulations confirmed stable binding of the CXCR2-Navarixin complex. qRT-PCR confirmed significant downregulation of all three genes in IPAH patients.

**Conclusion:**

This hypothesis-generating study identifies shared transcriptomic signatures between IPAH and MPM, highlighting CXCR2, S100A9, and LCN2 as candidate biomarkers linked to immune dysregulation and metabolic reprogramming. The computational and correlative nature of these findings, together with the limited size of the external validation cohort, requires confirmation through functional experiments and validation in larger, independent, and multicentre cohorts before clinical translation can be considered.

## Graphical Abstract

**Figure:**
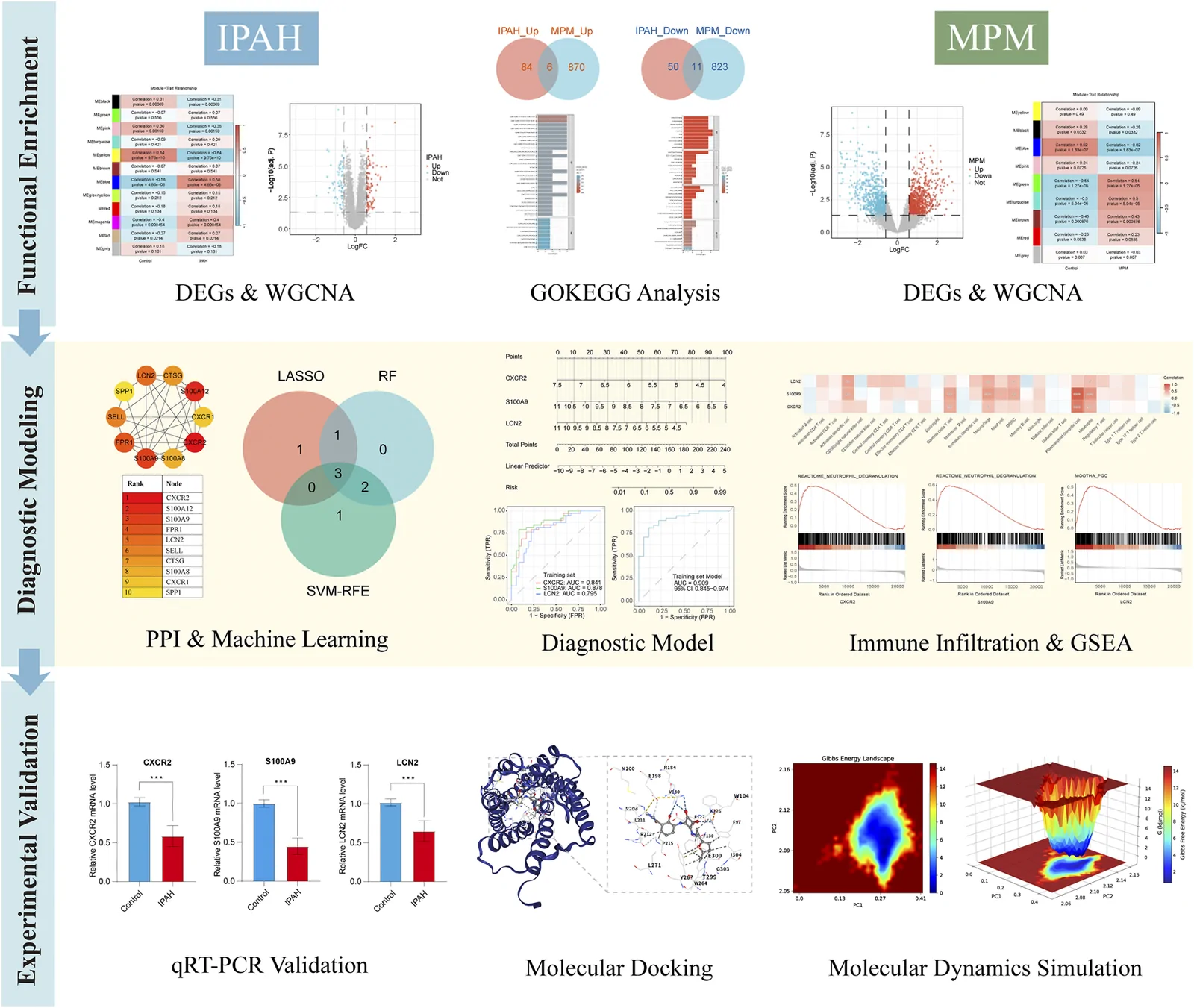

## 1 Introduction

Idiopathic pulmonary arterial hypertension (IPAH) is a lethal condition of the cardiovascular system marked by progressive remodeling of the pulmonary vasculature, eventually resulting in right-sided heart failure and death (Ruopp and Cockrill, 2022). Although targeted vasodilatory therapies, including endothelin receptor antagonists, phosphodiesterase-5 inhibitors, and prostacyclin analogs, have improved hemodynamic profiles in some patients (Liu et al., 2021; Zeng et al., 2023), these agents fail to reverse the underlying pathological vascular remodeling. Consequently, a substantial proportion of patients experience disease progression with persistently high mortality, and lung transplantation remains the only curative option available (Ko and laitis, 2023; Vicaire et al., 2022). This clinical impasse underscores a critical scientific question: the fundamental molecular mechanisms driving IPAH progression remain largely elusive.

Notably, compelling biological parallels exist between IPAH and malignancies. As early as 1998, Voelkel and colleagues proposed the “cancer hypothesis” for PAH (Voelkel et al., 1998), a concept that has since gained support from multiple lines of evidence. At the cellular phenotype level, remodeling cells in the pulmonary vasculature of IPAH patients, such as pulmonary arterial smooth muscle cells and endothelial cells, display characteristic features reminiscent of cancer cells. These include uncontrolled proliferation, resistance to apoptosis, and a shift in metabolism toward aerobic glycolysis (the Warburg effect) (Masri et al., 2007; Perros et al., 2019; Liu et al., 2022a) At the clonal level, plexiform lesions not only morphologically resemble tumor vasculature but also harbor endothelial cells of monoclonal origin (Lee et al., 1998). At the genetic level, oncogenic somatic mutations have been reported in plexiform lesions from an IPAH patient, further substantiating their neoplastic nature (Schimmel et al., 2026). Collectively, these observations indicate that pulmonary artery remodeling cells in IPAH display a distinct cancer-like phenotype.

Malignant pleural mesothelioma (MPM) is a rare but exceptionally aggressive tumor originating from pleural mesothelial cells (Khosla et al., 2024). Despite their distinct tissue origins, MPM and IPAH share multiple molecular pathological features. The rationale for selecting this malignancy as a comparator is as follows. First, both involve aberrant activation of the HSP90 chaperone system. HSP90 is highly expressed in MPM tumor cells and is essential for their survival (Okamoto et al., 2008); similarly, mitochondrial HSP90 accumulates specifically in pulmonary arterial smooth muscle cells from IPAH patients (Boucherat et al., 2018). This chaperone system confers apoptosis resistance and stress tolerance by inhibiting Cyclophilin D-mediated apoptotic signaling while preserving mitochondrial energy metabolism (Kang et al., 2007). Second, both diseases feature dysregulated matrix remodeling. In MPM, MMP-2 activation is driven by upregulation of MT1-MMP and downregulation of TIMP-2, promoting tumor invasion and angiogenesis (Zhong et al., 2006); notably, the same MMP-2 activation pathway is centrally involved in the pulmonary vascular remodeling seen in IPAH (Lepetit et al., 2005), with MMP-2 activity correlating positively with disease severity (Arvidsson et al., 2022). Third, unlike epithelial-derived malignancies, MPM and IPAH share a mesenchymal lineage cellular context. MPM originates from mesothelial cells, while IPAH vascular remodeling involves smooth muscle cell/fibroblast proliferation and endothelial-to-mesenchymal transition (Chen et al., 2026a). Additionally, both diseases have been reported to be associated with asbestos exposure (Janes et al., 2021; Wright et al., 1991). These unique commonalities in lineage context and environmental background make MPM a more relevant comparator than epithelial-derived cancers for investigating the cancer-like features of IPAH.

Building on this evidence, we propose a working hypothesis: IPAH may represent not merely a vascular disease but rather a condition with cancer-like features, characterized by aberrant proliferation and immune microenvironment remodeling. In this research, we combined publicly available transcriptomic datasets from IPAH and MPM patients to systematically explore their molecular associations. Using a multi-strategy approach including differential expression analysis, weighted gene co-expression network analysis (WGCNA), machine learning, immune infiltration profiling, molecular docking, molecular dynamics (MD) simulations, and qRT-PCR validation in human peripheral blood, we sought to (1) identify candidate biomarker genes shared between IPAH and MPM; (2) characterize the biological roles and immune microenvironment properties linked to these genes; (3) evaluate their potential as candidate biomarkers for IPAH; and (4) predict candidate drugs targeting these candidate biomarker genes. Through these analyses, we aim to generate hypotheses and provide a molecular foundation for the cancer-like hypothesis of IPAH, offer new insights into the pathological mechanisms underlying refractory IPAH, and suggest new directions for therapeutic development.

## 2 Materials and methods

### 2.1 Transcriptome data preprocessing

This research acquired gene expression profile data from the NCBI Gene Expression Omnibus (GEO) database (https://www.ncbi.nlm.nih.gov/geo/) (Barrett et al., 2005). A summary of all GEO datasets used in this study, including accession number, sample size, tissue source, platform, and specific purpose within the analytical workflow, is provided in Table 1. For IPAH, we included 6 cases and 11 controls from GSE113439, along with 32 cases and 25 controls from GSE117261. For MPM, the analysis encompassed 40 MPM cases and 5 controls from GSE2549, and 9 MPM cases and 4 controls from GSE12345. After merging the IPAH and MPM datasets separately, batch correction was performed on the combined datasets using the Combat method in the sva package (3.50.0 version) in R software (4.3.3 version) to remove batch effects.

**TABLE 1:** Summary of GEO datasets used in this study.

Accession	Sample size (case/control)	Tissue source	Platform	Purpose in analytical workflow
GSE113439	6/11	Lung tissue	GPL6244	IPAH DEG identification
GSE117261	32/25	Lung tissue	GPL6244	IPAH DEG identification
GSE2549	40/5	Pleural tissue	GPL96	MPM DEG identification
GSE12345	9/4	Pleural tissue	GPL570	MPM DEG identification
GSE130391	4/4	Pulmonary artery	GPL570	Independent external validation

### 2.2 Differential expression gene analysis

To detect overlapping genes and common molecular mechanisms between IPAH and MPM, we conducted a differential expression analysis using the combined datasets. Following batch correction, case and healthy control samples were compared via differential expression analysis using version 3.58.1 of the limma package (Ritchie et al., 2015). An empirical Bayes approach was employed for this analysis. For both IPAH and MPM, genes were classified as differentially expressed if they met two criteria: an adjusted P-value less than 0.05 and an absolute log2 fold change greater than 0.585. This threshold corresponds to |log2 (1.5)|, representing a 1.5-fold change cutoff that balances sensitivity for detecting moderate but biologically meaningful expression changes against stringency for excluding noise in microarray-based transcriptomic comparisons. For data visualization, version 3.5.2 of ggplot2, version 0.6.0 of ggpubr, and version 1.0.12 of pheatmap were employed for graphical display of DEG expression patterns. The intersection of upregulated (or downregulated) DEGs between IPAH and MPM was defined as the concordantly deregulated gene sets for subsequent analysis.

Because the combined MPM cohort comprised 49 cases and only 9 controls (approximately 5.4:1 ratio), a sensitivity analysis was performed using the Reproducibility-Optimized Test Statistic (ROTS) method, a non-parametric approach robust to sample size imbalance (Suomi et al., 2017). ROTS was run on the same batch-corrected expression matrix with B = 1,000 bootstrap iterations, K = 500, and seed = 123. The primary ROTS comparison used FDR <0.05, consistent with the limma threshold (adjusted *P* < 0.05) used to define the DEG list, with FDR <0.10 included as a more permissive sensitivity check. Direction concordance was defined as the proportion of overlapping genes with the same sign of log fold change between limma and ROTS. A permutation test (1,000 iterations) was used to estimate the overlap expected by chance.

### 2.3 Gene ontology and pathway enrichment analysis

To explore the biological functions of the concordant DEGs, we performed enrichment analyses using the clusterProfiler package (v4.10.1). Specifically, Gene Ontology (GO) (Ashburner et al., 2000) and Kyoto Encyclopedia of Genes and Genomes (KEGG) (Kanehisa and Goto, 2000) analyses were carried out separately on the upregulated and downregulated gene sets. GO analysis elucidated the associated biological processes (BP), molecular functions (MF), and cellular components (CC). KEGG analysis was conducted to identify significantly enriched signaling pathways. The cutoff for enrichment significance was defined as *P* < 0.05.

### 2.4 Weighted gene co-expression network analyses

To establish co-expression networks and detect gene modules linked to the disease, separate WGCNA was done for IPAH and MPM (Langfelder and Horvath, 2008). Input matrices were constructed using all genes from each combined dataset. Soft thresholding values of 1–20 were applied to choose the optimal cutoff for network analysis. The correlation matrix was used to compute an adjacency matrix, which was then transformed to generate the topological overlap matrix (TOM). Similar module clusters were identified via average linkage hierarchical clustering of the TOM and merged together. For each disease, the single module showing the highest correlation with the disease phenotype was selected using Pearson correlation coefficients. Gene significance (GS) was derived from the correlation between each gene’s expression profile and the disease trait. Module membership (MM), defined as the correlation between a gene’s expression profile and the module eigengene, was used to measure a gene’s connectivity within a module.

### 2.5 Protein-protein interaction (PPI) network building

The combination of concordant DEGs (Section 2.2) and genes of core WGCNA modules (Section 2.4) with duplicates removed generated the PPI network with 72 unique candidate genes. These candidate genes were submitted to the STRING database (https://string-db.org/) to retrieve PPI information (Szklarczyk et al., 2025). Cytoscape (version 3.9.1) was used to visualize the network and identify the most connected genes (Shannon et al., 2003). The top 10 candidate genes showing the strongest connectivity within the PPI network were detected using the MCC method from the CytoHubba plugin.

### 2.6 Machine learning for candidate biomarker gene screening

LASSO regression offers advantages in feature shrinkage and interpretability, Random Forest captures non-linear interactions and ranks feature importance, while SVM-RFE excels at iterative feature elimination in high-dimensional data. To screen for candidate biomarker genes for IPAH from the candidate genes in the PPI network, we therefore jointly used these three algorithms. The top 10 candidate gene expression levels were used as features, with disease status as the response variable. Using the caret package (version 7.0–1), we randomly split the IPAH dataset into training and testing sets at a 7:3 ratio.

Several strategies were employed to minimize overfitting throughout the modeling process. For LASSO regression (glmnet package, version 4.1–8), 10-fold cross-validation was used to determine the optimal regularization parameter λ, with the value corresponding to lambda. min selected. The Random Forest model (randomForest package, version 4.7–1.2) used 500 trees with out-of-bag error estimation to assess generalization performance; the top 6 genes were selected according to the mean decrease in the Gini index. The SVM-RFE model (e1071 package, version 1.7–16) employed a recursive feature elimination approach with 10-fold cross-validation to guide the elimination process. All feature selection was performed exclusively on the training set, and model performance was subsequently evaluated on the held-out testing set and an independent external validation dataset (GSE130391) to assess generalizability. The ultimate candidate biomarker genes were identified as the overlap of genes chosen through all three approaches.

### 2.7 Classification model construction and validation

A classification model for IPAH was built using the three candidate biomarker genes, with the explicit purpose of hypothesis generation and mechanistic exploration rather than clinical diagnosis. To illustrate the impact of each gene on disease risk, we generated a nomogram via the rms package (version 6.7–1). ROC curve analysis was done to assess the discriminatory power of the model, with the AUC being computed via the pROC package (v1.18.5). We also performed independent ROC analyses for each biomarker gene to assess their individual discriminatory value. Finally, an independent dataset (GSE130391, n = 4 cases and 4 controls) was utilized to externally evaluate the model and individual gene performance. Given the very small size of this validation cohort, the AUC estimates, particularly the perfect AUC of 1.0 observed for LCN2, should be interpreted with caution because they may be inflated by batch effects, overfitting, or the small number of validation samples.

To assess potential overfitting, leave-one-out cross-validation (LOOCV) and bootstrap optimism correction (1,000 iterations) were additionally performed on the combined IPAH dataset (n = 74; 38 IPAH and 36 controls). For LOOCV, the classifier was trained on n−1 samples and tested on the held-out sample in each iteration. For bootstrap optimism correction, 1,000 bootstrap resamples were generated. In each resample, the model was refit, and the optimism was calculated as the difference between the AUC on the bootstrap sample and the AUC on the original dataset. The optimism-corrected AUC was defined as the apparent AUC minus the mean optimism.

### 2.8 Immune cell infiltration analysis

ssGSEA was conducted via the GSVA package (v1.50.5) in R to assess immune cell infiltration levels in IPAH cases and healthy controls. The enrichment scores of all 28 types of immune cells were determined for every sample based on preset signature gene sets. Spearman’s rank correlation test was used to correlate the expression of the three candidate biomarker genes with the infiltration scores of different immune cells. We visualized the resulting correlations as a heatmap using the corrplot package (version 0.95). It should be noted that immune cell infiltration was inferred using transcriptomic deconvolution methods rather than direct experimental measurements.

### 2.9 Single-gene differential analysis and pathway enrichment

We separated the specimens into elevated and reduced expression groups according to the midpoint value of every candidate biomarker gene’s expression. Gene Set Enrichment Analysis (GSEA) was conducted on all genes comparing the two groups within the IPAH dataset via the clusterProfiler package (v4.10.1) to identify signaling pathways that might be regulated by the candidate biomarker genes. Adjusted *P* < 0.05 was set as the enrichment significance cutoff.

### 2.10 Molecular docking

Candidate drugs targeting CXCR2, S100A9, and LCN2 were retrieved via the DGIdb database (https://www.dgidb.org/) (Cannon et al., 2024). The protein sequences of the target proteins were collected from the UniProt database (https://www.uniprot.org/) (Ahmad et al., 2025). Their three-dimensional X-ray crystal structures were retrieved from the RCSB Protein Data Bank (https://www.rcsb.org/) (Berman et al., 2000). The chemical structures of the candidate compounds were obtained from the PubChem database (https://pubchem.ncbi.nlm.nih.gov/) (Kim et al., 2025). PyMOL software (version 2.6.0a0) was used to preprocess the protein and ligand structures (Mooers, 2020). Molecular docking analysis between the candidate compounds and the target proteins was subsequently done using the CB-Dock tool (https://cadd.labshare.cn/cb-dock2/php/blinddock.php) (Liu et al., 2022b; Yang et al., 2022).

### 2.11 Molecular dynamics simulations

MD simulations were performed using GROMACS 2022.2 to investigate the dynamic behavior of the receptor–ligand complex (Abraham et al., 2015/09). The receptor force field parameters were established via the Amber99SB-ILDN force field (Maier et al., 2015), while those for the ligand were assigned via the GAFF2 force field using the sobtop_1.0 (dev3.1) tool (Wang et al., 2004). To ensure physicochemical consistency, partial charges of the ligand were determined with the RESP method. The system was then solvated in a TIP3P water box, maintaining a minimum distance of 1.0 nm between the protein atoms and the box edge to avoid periodic boundary artifacts. To achieve charge neutrality and simulate physiological ionic strength, Na+ and Cl-ions were added using the gmx genion tool of GROMACS 2022.2 to a final concentration of 0.15 M NaCl. Long-range electrostatic interactions were treated with the Particle Mesh Ewald (PME) method employing a cutoff distance of 1.0 nm, while bond constraints were handled using the LINCS algorithm. Prior to the production MD simulations, the system was subjected to energy minimization to remove steric clashes and unfavorable contacts. This minimization was performed sequentially using 3,000 cycles of steepest descent, then 2000 cycles of conjugate gradient for further refinement. Simulations were conducted in the NPT ensemble for 100 ns with an integration time step of 2 fs. The temperature was maintained at 310 K using a Nosé–Hoover thermostat (coupling constant: 0.1 ps), while the pressure was kept at 1 bar using a Parrinello–Rahman barostat (coupling constant: 2.0 ps) to maintain physiological conditions throughout the simulation. The following analyses were performed using the respective GROMACS tools: root-mean-square deviation (RMSD, gmx rmsd), root-mean-square fluctuation (RMSF, gmx rmsf), number of hydrogen bonds (gmx hbond), radius of gyration (Rg, gmx gyrate), solvent-accessible surface area (SASA, gmx sasa), Gibbs free energy landscape (gmx sham), and distances between specific atoms of key residues and the ligand (gmx distance). These analyses were used to evaluate system stability, conformational changes, and solvent effects.

### 2.12 Ethics statement and patient selection

Approval for this retrospective study was granted by the Institutional Review Board of the First Affiliated Hospital of Xinjiang Medical University on 19 March 2026 (Approval No. K202603-76). The ethics committee exempted the need for written informed consent, as this research used residual peripheral blood samples from the clinical laboratory that were collected prior to the approval date, with all samples de-identified.

Patients with IPAH were identified by searching the hospital’s electronic health record database at the First Affiliated Hospital of Xinjiang Medical University between 1 December 2025 and 1 March 2026. Inclusion criteria were: (1) diagnosis of IPAH confirmed by right heart catheterization according to the 2022 ESC/ERS Guidelines (Humbert et al., 2022); (2) availability of residual peripheral blood samples in the clinical laboratory. Exclusion criteria were: (1) secondary pulmonary arterial hypertension (e.g., associated with connective tissue disease, congenital heart disease, or portal hypertension); (2) concomitant severe hepatic or renal dysfunction; (3) active infection or malignancy. Healthy controls were selected from persons who had routine health check-ups within the same timeframe, with no history of cardiovascular or pulmonary diseases. A total of 8 IPAH patients and 8 healthy controls were included in this study. We acknowledge that this limited sample size may affect the statistical robustness and generalizability of the validation results.

### 2.13 Real-time quantitative reverse transcription PCR

Peripheral blood samples from the 8 healthy controls and 8 patients with IPAH described above served as independent biological replicates. About 5 mL of venous blood was drawn from every subject and immediately frozen for storage at −80 °C until RNA extraction. Total RNA was isolated with TRIzol™ Reagent (Cat# 15596026, Invitrogen, United States) according to the manufacturer’s instructions. The RNA yield was approximately 20 μg per sample. The A260/A280 ratio was measured using a NanoDrop spectrophotometer to determine RNA purity, with only samples exhibiting ratios between 1.9 and 2.1 being retained for further analysis. RNA integrity was assessed using an Agilent 2,100 Bioanalyzer (Agilent Technologies, United States), with RIN values ranging from 7.5 to 9.2, and further verified by agarose gel electrophoresis, where the presence of clear 28S and 18S ribosomal RNA bands (28S/18S ratio≥2.0) confirmed intact RNA. To exclude genomic DNA contamination, no-RT controls (omitting reverse transcriptase) were performed for each sample.

Reverse transcription was performed using 1 µg of total RNA in a 20 µL reaction volume with the Transcriptor First Strand cDNA Synthesis Kit (Cat# 04 379 012 001, Roche, Switzerland). The reaction mixture contained random hexamer primers (2.5 μM) and Transcriptor Reverse Transcriptase (10 U/μL). The reaction conditions were: 25 °C for 10 min, 50 °C for 60 min, and 85 °C for 5 min. The obtained cDNA was stored at −20 °C until use.

qRT-PCR was done using SYBR Green Master Mix (Roche, Switzerland) via an ABI StepOnePlus Real-Time PCR System (Applied Biosystems, United States) in a final volume of 20 µL containing 2 µL of 1:5 diluted cDNA, 0.4 µM each of forward and reverse primers, and 10 µL of 2× SYBR Green Master Mix. The thermal cycling protocol consisted of 95 °C for 10 min, then 40 cycles of 95 °C for 15 s and 60 °C for 60 s. A dissociation curve analysis was performed from 60 °C to 95 °C with a heating rate of 0.5 °C per 5 s to confirm single-product amplification. Each sample was run in triplicate technical replicates. No-template controls (NTC) were included in each run and showed no amplification (Cq > 40), confirming no contamination.

PCR efficiency for each target gene (CXCR2, S100A9, LCN2) and the reference gene ACTB was determined from standard curves generated by 5-fold serial dilutions of pooled cDNA. The coefficient of determination (r^2^) of the standard curves was 0.992 for all genes. Relative gene expression was calculated using the 2^−ΔΔCT^ method with ACTB as the internal control. ACTB was used as a single reference gene based on previous studies and its stable expression under the experimental conditions used. For each biological replicate (individual sample), the ΔCt was first calculated as (Ct_target–Ct_ACTB). The ΔΔCt was then obtained by subtracting the mean ΔCt of the control group from the ΔCt of each IPAH sample. The fold change for the IPAH group relative to the control group is presented as mean ± SEM.

Primer specificity was confirmed as follows. First, an *in silico* specificity screen using BLAST against the human reference genome showed no off-target amplification of pseudogenes or homologs. Second, secondary structure analysis of the amplicons was performed using Primer-BLAST and Mfold, confirming minimal secondary structure at the reaction temperatures. Third, primers were designed to target conserved exon boundaries present in all validated transcript variants listed in NCBI RefSeq, ensuring universal detection of splice variants. GenBank accession numbers: NM_001168298.2 for CXCR2, NM_002965.4 for S100A9, NM_005564.5 for LCN2; amplicon lengths: 101 bp, 80 bp, and 137 bp, respectively. All oligonucleotide primers were synthesized by Sangon Biotech (Shanghai) Co., Ltd. (Shanghai, China), purified by PAGE, and supplied as lyophilized powder (no buffer). No probes were used (SYBR® Green). No chemical modifications were introduced. Primer sequences are shown in Table 2.

**TABLE 2:** Gene primer sequences.

Gene	Forward primer (5′-3′)	Reverse primer (5′-3′)
CXCR2	CTG​GGT​ACA​GTG​CTA​TTC​TGC	GCA​GCT​GTG​ACC​TGC​TGT​TAT
S100A9	AGT​GGC​CAA​GAT​CAC​AGT​GG	GCC​TGG​CCT​CCT​GAT​TAG​TG
LCN2	GGG​AGA​ACC​AAG​GAG​CTG​AC	CGGCACCTGTGCACTCA
ACTB	CCC​GCG​AGT​ACA​ACC​TTC​TT	CGC​AGC​GAT​ATC​GTC​ATC​CA

### 2.14 Statistical analysis

All data processing and statistical analyses involved in this study were done via R software (Version 4.3.3) and GraphPad Prism (Version 10.1.2). To compare continuous variables across two groups, the Shapiro–Wilk test was applied to evaluate data normality. For data with a normal distribution, the Student’s t-test was used; for data with a non-normal distribution, the Mann-Whitney U test was used. A two-tailed P-value below 0.05 was regarded as statistically significant.

## 3 Results

### 3.1 Screening of differentially expressed genes in IPAH and MPM

In the combined IPAH dataset (GSE113439 and GSE117261), 151 differentially expressed genes (DEGs) were initially identified, including 90 upregulated and 61 downregulated genes (Figure 1A). In the combined MPM dataset (GSE2549 and GSE12345), 1710 DEGs were identified, including 876 upregulated and 834 downregulated genes (Figure 1C). Heatmaps displayed the top 10 significantly upregulated and downregulated genes for IPAH and MPM, respectively (Figures 1B,D).

**FIGURE 1:**
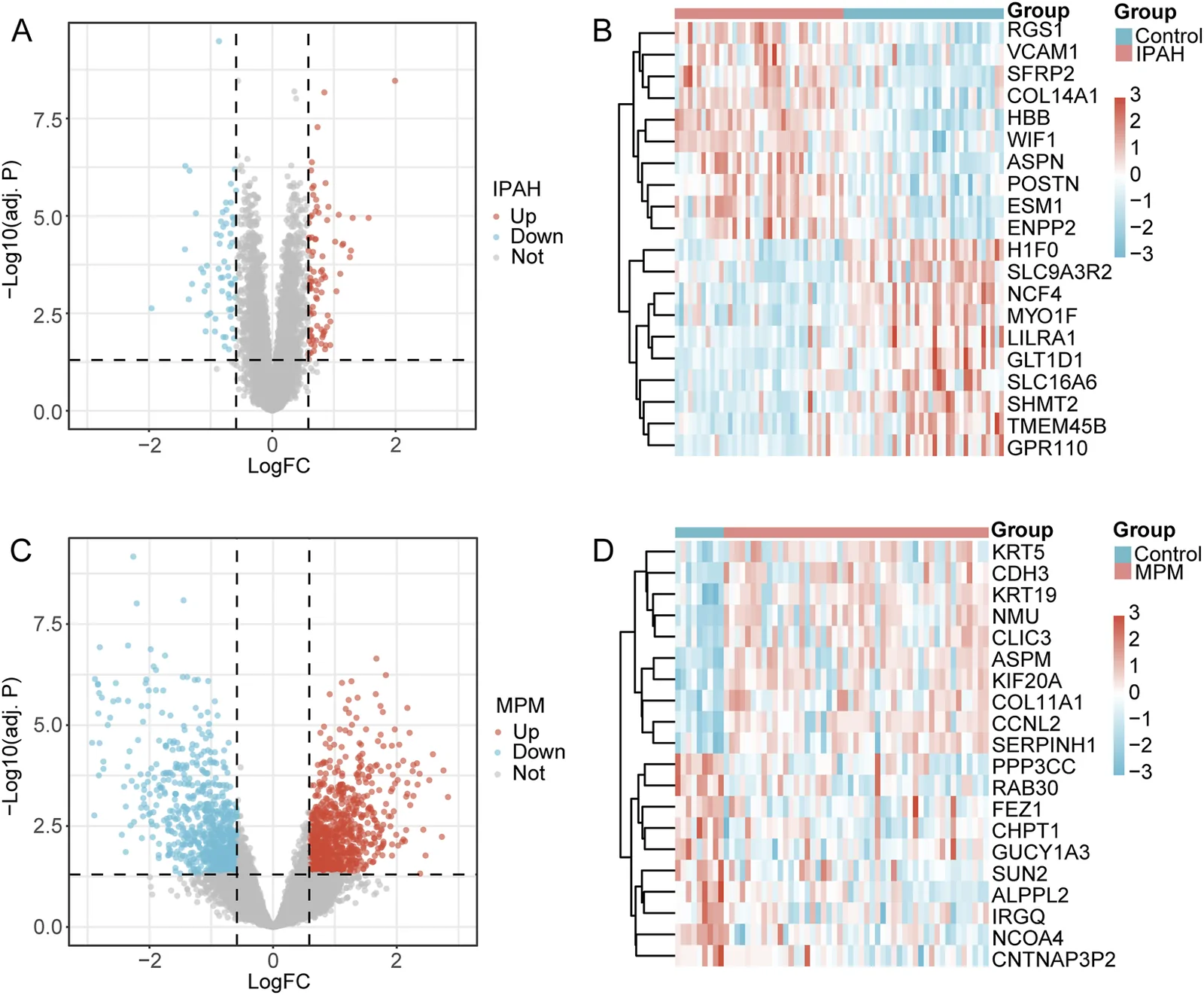
Identification of DEGs in IPAH and MPM. **(A)** Volcano plot of DEGs in the integrated IPAH cohort (GSE113439 and GSE117261). **(B)** Heatmap of the top 10 DEGs in the integrated IPAH cohort. **(C)** Volcano plot of DEGs in the integrated MPM cohort (GSE2549 and GSE12345). **(D)** Heatmap of the top 10 DEGs in the integrated MPM cohort.

Because the MPM cohort contained far fewer controls than cases (9 versus 49), we evaluated the stability of the limma-derived MPM DEG list using a ROTS sensitivity analysis. At the matching FDR <0.05 threshold, all 1710 limma-identified DEGs were among the ROTS-significant genes, with 100% direction concordance (Table 3), and results were consistent at FDR <0.10. A permutation test (1,000 iterations) showed that the overlap expected by chance was only 233 genes (95th percentile: 254), corresponding to a 7.3-fold enrichment over random expectation. These results indicate that the MPM DEG list was highly reproducible with a non-parametric approach and was not materially driven by the case–control imbalance.

**TABLE 3:** ROTS sensitivity analysis of MPM DEGs.

FDR threshold	ROTS significant genes	Overlap with limma DEGs	Overlap rate	Direction concordance
0.05	2,308	1710	100%	100%
0.10	3,298	1710	100%	100%

The primary comparison was performed at FDR <0.05 to match the limma threshold (adjusted *P* < 0.05). FDR <0.10 was included as a more permissive sensitivity check.

### 3.2 Enrichment analysis of concordant deregulated genes in IPAH and MPM

Overlap assessment of DEGs from IPAH and MPM revealed 6 concordantly upregulated genes and 11 concordantly downregulated genes, defined as genes differentially expressed in the same direction in both diseases (Figures 2A, 3A). We performed GO and KEGG enrichment analyses to explore the biological functions of these concordant genes. The enrichment analysis results exhibited that the concordantly upregulated genes in IPAH and MPM were primarily involved in processes such as negative regulation of the G1/S phase transition of the cell cycle (Figure 2B), with no significantly enriched KEGG pathways identified. In contrast, the concordantly downregulated genes were markedly enriched in immune-inflammatory pathways associated with neutrophil chemotaxis and migration, immune receptor activity, and neutrophil extracellular trap formation (Figure 3B).

**FIGURE 2:**
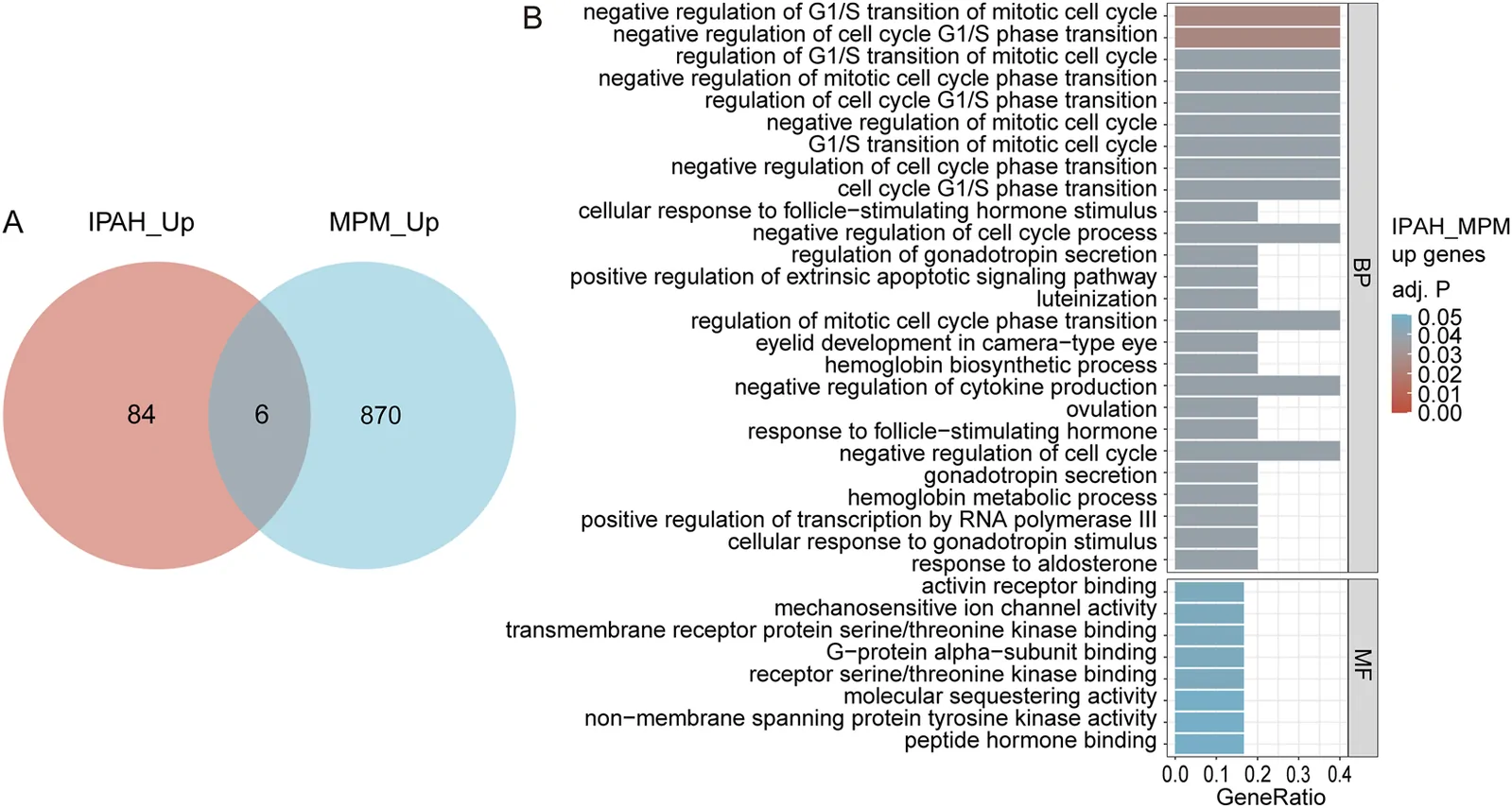
GO analysis of concordantly upregulated DEGs in IPAH and MPM. **(A)** Venn diagram showing the overlap of upregulated DEGs between IPAH and MPM. **(B)** GO analysis of the 6 concordantly upregulated genes shared by IPAH and MPM. No significant enrichment of KEGG pathways was identified for this gene set.

**FIGURE 3:**
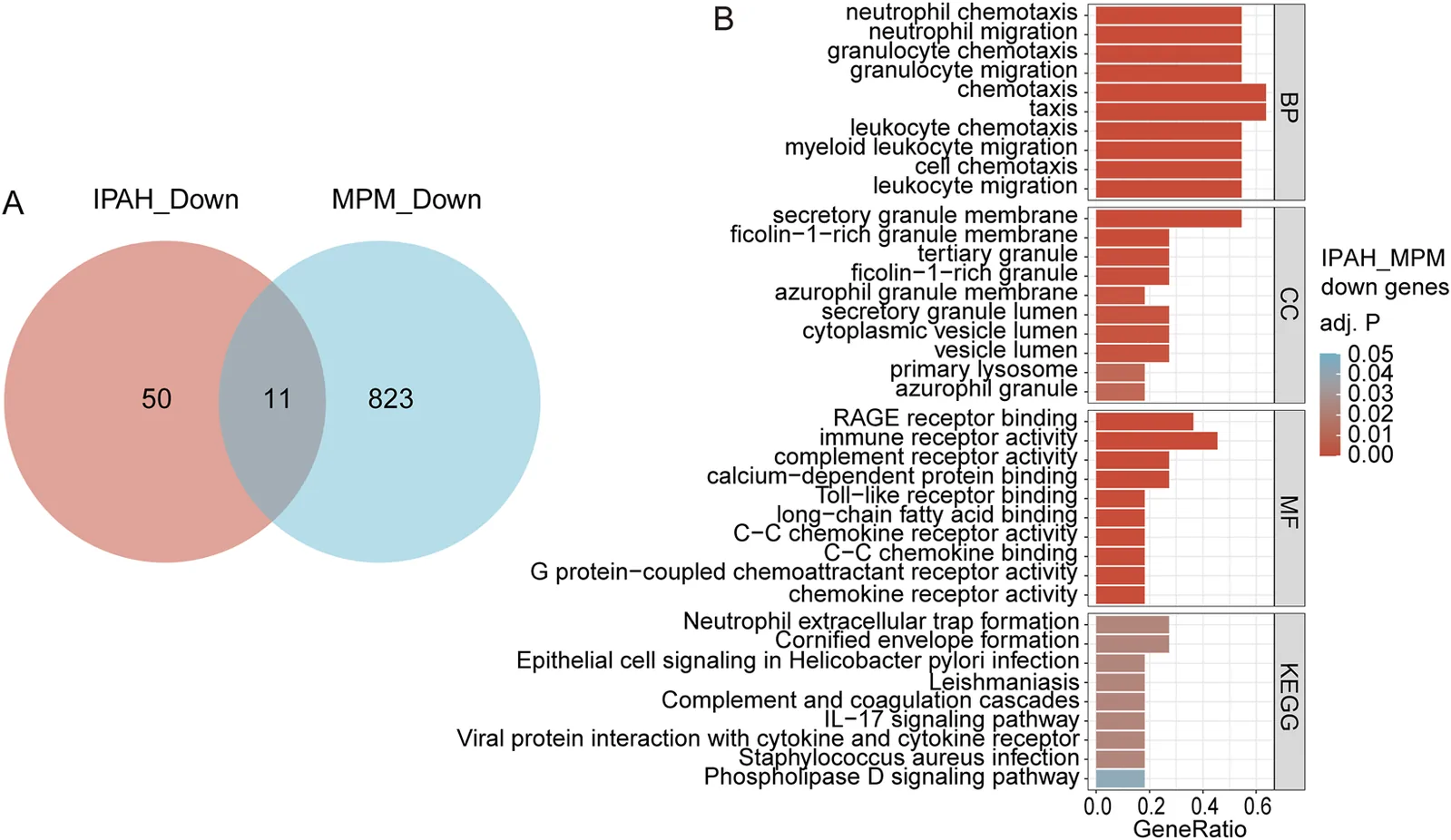
GO and KEGG analysis of concordantly downregulated DEGs in IPAH and MPM. **(A)** Venn diagram showing the overlap of downregulated DEGs between IPAH and MPM. **(B)** GO and KEGG pathway analysis of the 11 concordantly downregulated genes shared by IPAH and MPM.

### 3.3 Weighted gene co-expression network analysis of IPAH and MPM

A separate WGCNA was performed on the combined datasets for IPAH and MPM. The optimal soft thresholding power was determined as the first value reaching the scale-free topology fit threshold: β = 5 (R2 = 0.8) for IPAH and β = 3 (R2 = 0.9) for MPM (Figures 4A,B). In the IPAH dataset, 12 gene modules were detected, and the yellow module exhibited the highest negative association with the IPAH phenotype (r = −0.64; Figure 4C). In the MPM dataset, 9 gene modules were uncovered, and the blue module displayed the strongest negative correlation with the MPM phenotype (r = −0.62; Figure 4D). A strong correlation between gene significance (GS) and module membership (MM) was observed within the yellow module of IPAH and the blue module of MPM (Figures 4E,F). Intersection analysis of the genes in these two core modules revealed 58 overlapping genes, which were defined as the shared core module genes for IPAH and MPM (Figure 4G).

**FIGURE 4:**
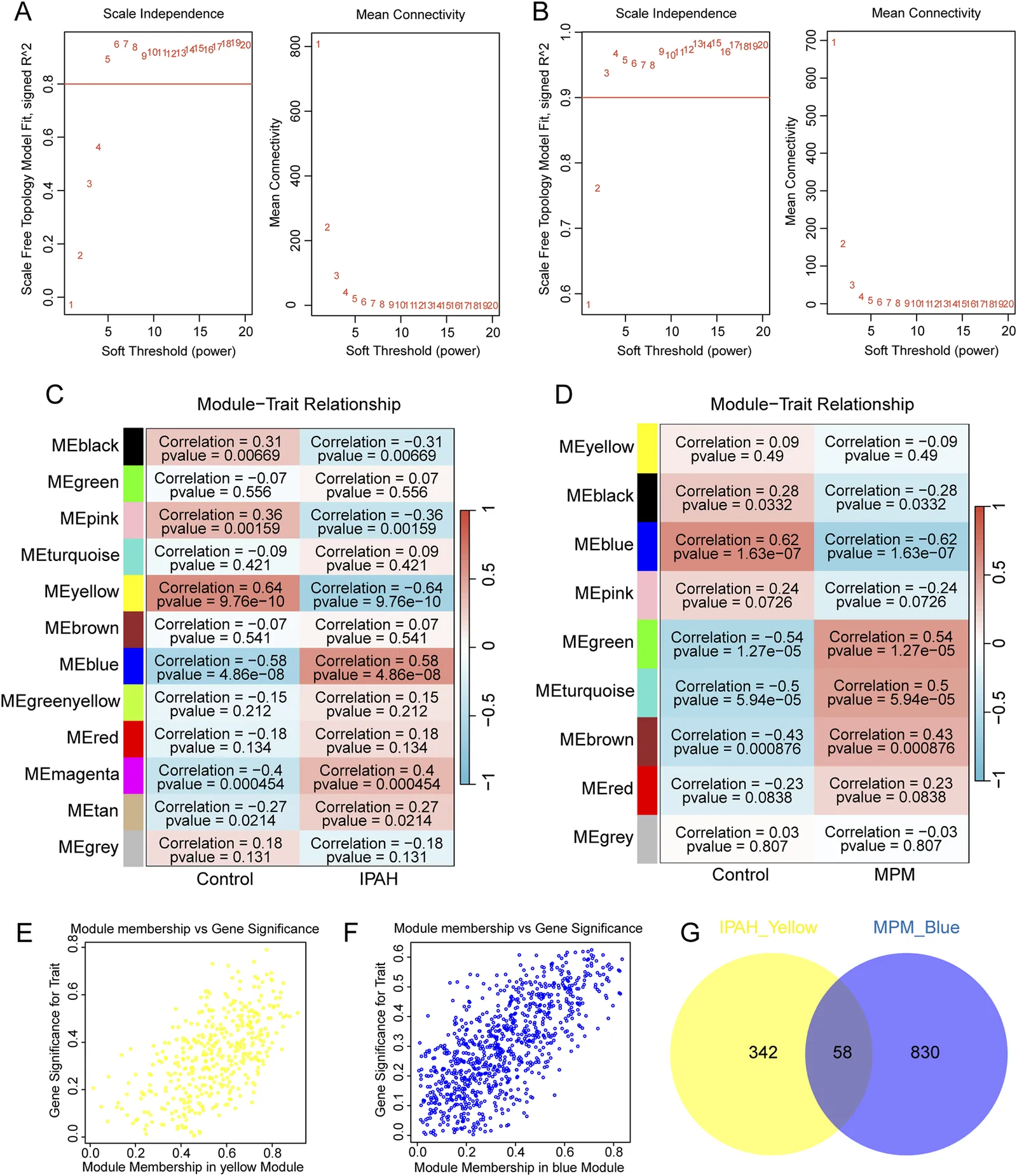
WGCNA analysis of IPAH and MPM. **(A,B)** Scale independence (left) and mean connectivity (right) as functions of the soft-thresholding power (β) in the integrated IPAH cohort and the integrated MPM cohort. **(C,D)** Heatmaps showing correlations between module eigengenes and disease phenotypes in IPAH and MPM, with red indicating positive correlations and blue indicating negative ones. **(E,F)** GS versus MM in the yellow module of IPAH and the blue module of MPM. **(G)** Venn diagram illustrating overlapping genes between the IPAH yellow module and the MPM blue module.

### 3.4 Protein-protein interaction network analysis

To avoid missing potential key genes, we merged the 17 concordant genes from differential analysis with the 58 shared core module genes from WGCNA. After removing duplicates, 72 candidate genes were obtained. A PPI network was built via the STRING database based on these 72 genes (Figure 5A). Then, the MCC method in CytoHubba was employed to screen the top 10 candidate genes: CXCR2, S100A12, S100A9, FPR1, LCN2, SELL, CTSG, S100A8, CXCR1, and SPP1 (Figure 5B). Although these genes were derived from the intersection of IPAH and MPM, subsequent analyses focused on IPAH to explore its classification value and cancer-like features.

**FIGURE 5:**
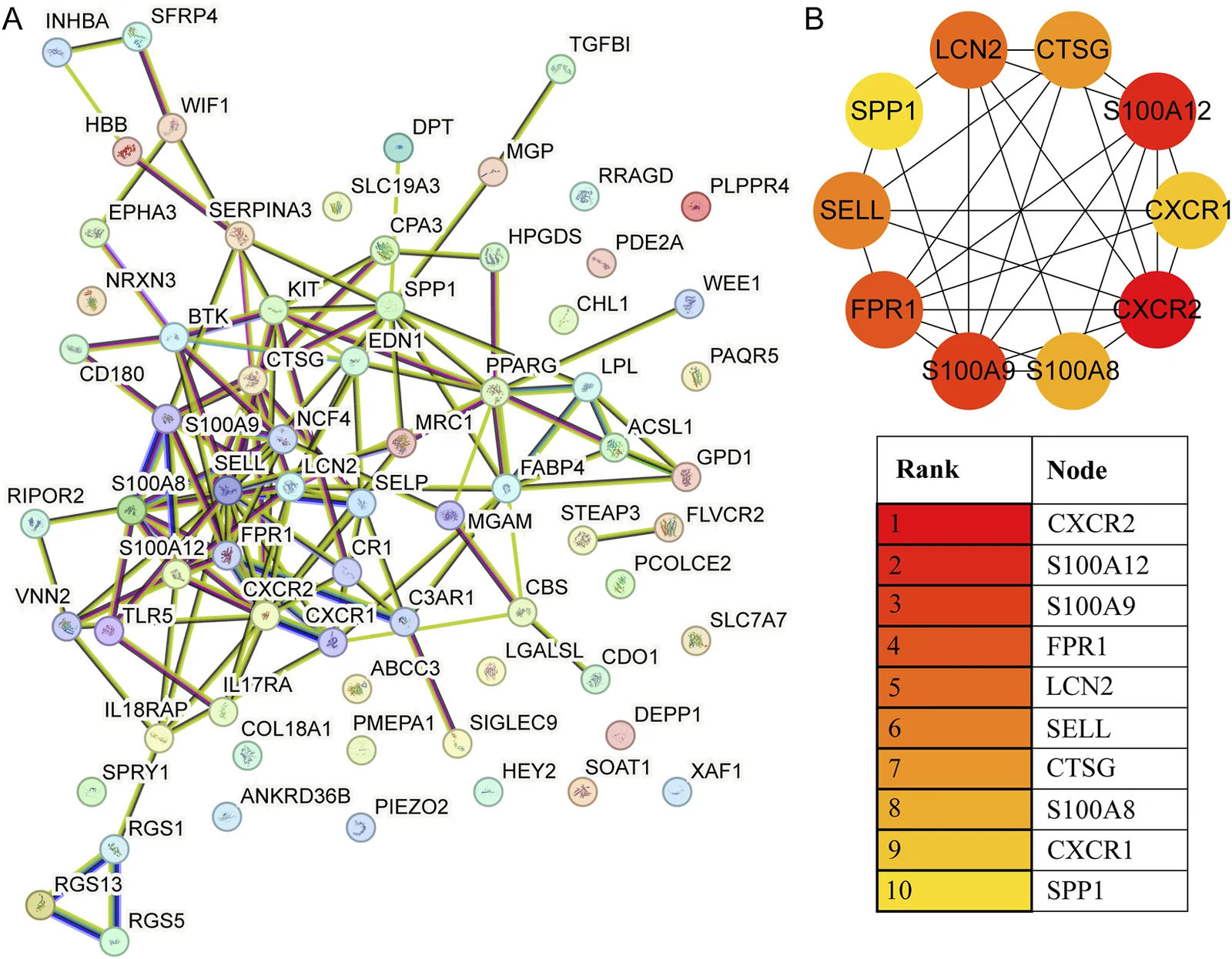
Identification of candidate genes via PPI network. **(A)** PPI network of the 72 candidate genes. **(B)** Top 10 candidate genes identified from the PPI network using the MCC algorithm.

### 3.5 Screening of candidate biomarker genes via machine learning

To further pinpoint candidate biomarker genes for IPAH among the candidate genes, we applied three machine learning approaches to perform feature selection using the expression of the 10 candidate genes as input: LASSO, RF, and SVM-RFE. The optimal regularization parameter λ for LASSO was determined by ten-fold cross-validation (Figure 6A). LASSO regression retained 5 genes: CXCR2, S100A9, LCN2, CTSG, and SPP1, with coefficient paths shown in Figure 6B. RF selected the top 6 genes based on the mean decrease in the Gini index: CXCR2, S100A9, CXCR1, S100A8, SELL, and LCN2. SVM-RFE independently identified the same set of 6 genes, demonstrating the robustness of these features across different algorithms. The feature importance and residual diagnostics for both models are presented in Figures 6C–E. The common genes simultaneously picked by all three algorithms comprised CXCR2, S100A9, and LCN2 (Figure 6F). Therefore, these three genes were established as the candidate biomarker genes for IPAH and used for subsequent model construction.

**FIGURE 6:**
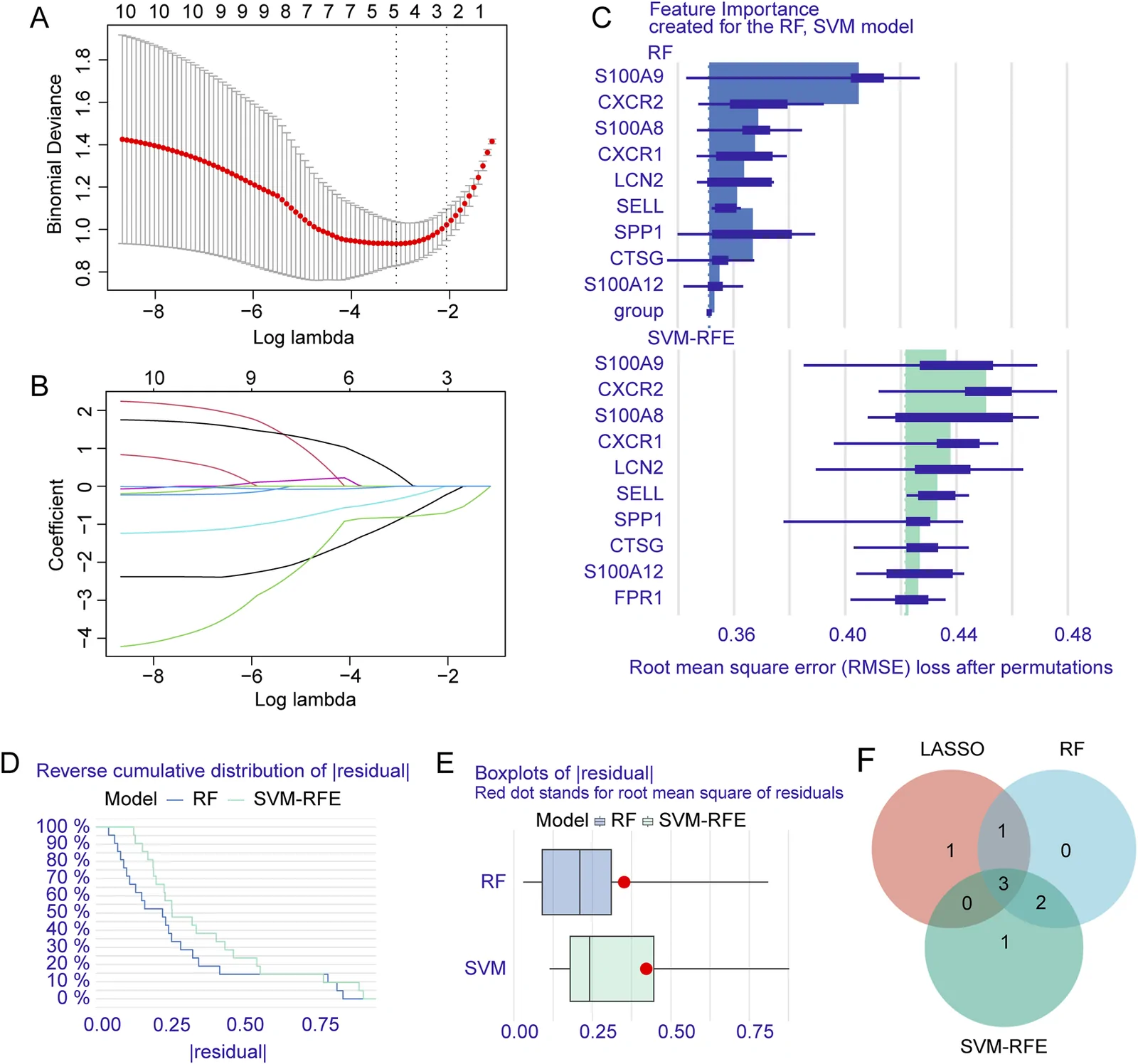
Screening of candidate biomarker genes by multiple machine learning algorithms. **(A)** Cross-validation for tuning the LASSO regularization parameter (λ). **(B)** LASSO coefficient paths of the 10 candidate genes. **(C)** Feature importance plots from the RF and SVM-RFE algorithms. **(D)** Cumulative distribution (reverse) of absolute forecast errors for the RF and SVM-RFE approaches. **(E)** Boxplots of absolute residuals for the RF and SVM-RFE models. **(F)** Overlap visualization of candidate biomarker genes commonly identified by LASSO, RF, and SVM-RFE.

### 3.6 Classification model construction and validation

A classification model for IPAH was constructed based on the three candidate biomarker genes CXCR2, S100A9 and LCN2. The nomogram visually depicted each gene’s contribution to IPAH risk (Figure 7A). The model’s net clinical value and predictive performance were assessed via decision curve analysis (DCA) alongside calibration plots (Figures 7B,C). In the training set, the independent classification AUC values for the three genes were 0.841 for CXCR2, 0.878 for S100A9, and 0.795 for LCN2 (Figure 7D). The combined model achieved an AUC of 0.909 (Figure 7E). In the independent validation set GSE130391 (n = 4 cases and 4 controls), the individual AUC values for the three genes were 0.688 for CXCR2, 0.812 for S100A9, and 1.000 for LCN2 (Figure 7F). The combined model yielded an AUC of 0.875 when tested on the external validation cohort (Figure 7G). These findings indicate that the classification model exhibits encouraging predictive performance. However, the perfect AUC of 1.0 for LCN2 in the validation set is likely to reflect the very small number of validation samples (n = 4 per group) and possible batch effects between the training and validation datasets rather than genuine biological separation; these estimates should be considered exploratory and hypothesis-generating, and validation in larger, independent, and multicentre cohorts is necessary.

**FIGURE 7:**
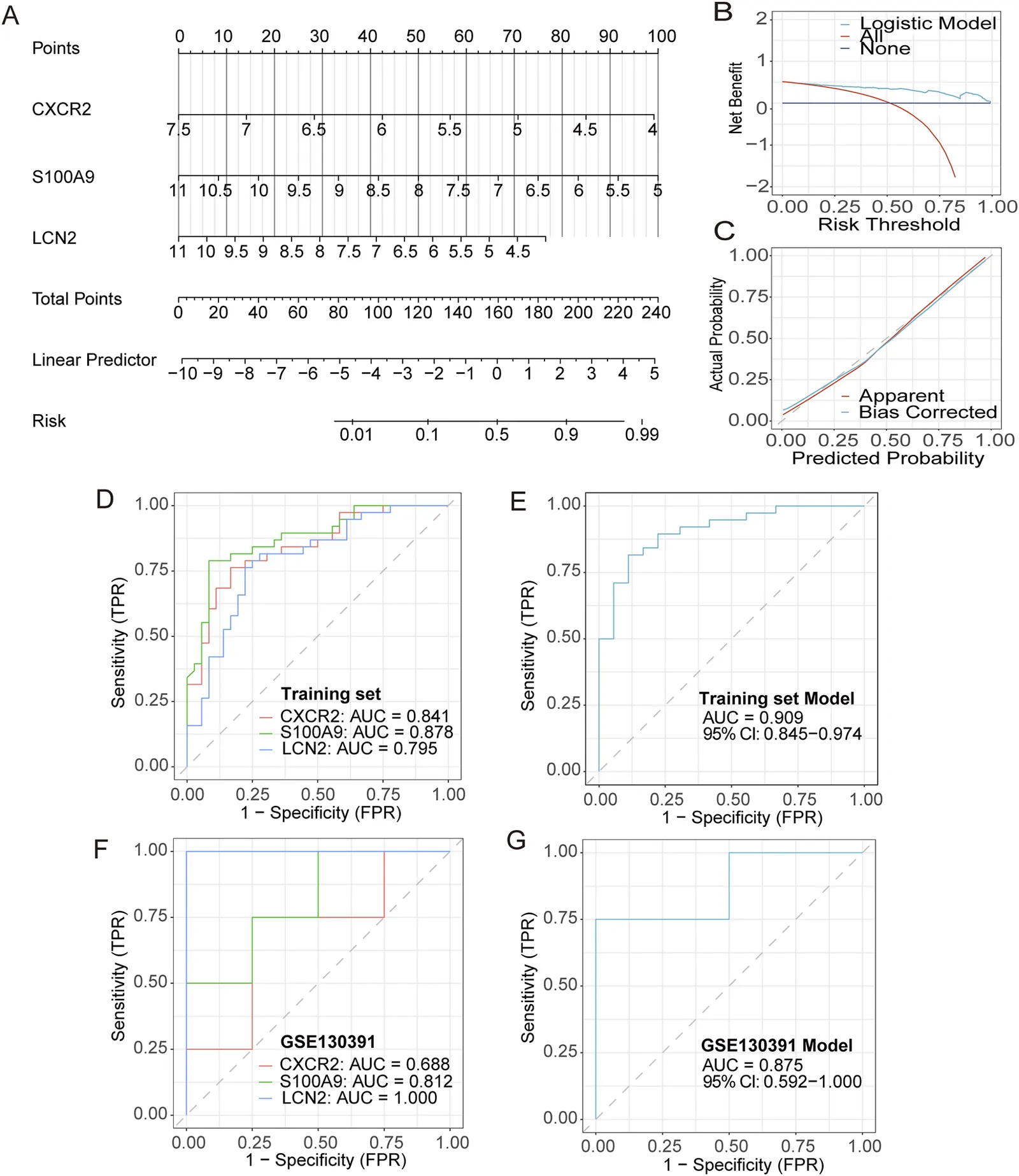
Construction and validation of the classification model for IPAH. **(A)** Nomogram for predicting IPAH probability based on CXCR2, S100A9, and LCN2 expression. **(B)** DCA of the classification model. **(C)** Calibration curve of the classification model. **(D)** ROC curves for individual genes in the training set. **(E)** ROC curve of the combined transcriptomic signature based on three genes in the training cohort. **(F)** ROC curves of single genes within the external validation dataset (GSE130391). **(G)** ROC curve for the three-gene combined model in the validation set.

To assess potential overfitting, we performed LOOCV and bootstrap optimism correction (1,000 iterations) on the combined IPAH dataset (n = 74; 38 IPAH and 36 controls) (Table 4). For the combined three-gene classifier, the apparent AUC was 0.909 (95% CI: 0.845–0.974), and the LOOCV AUC was 0.879 (95% CI: 0.801–0.957), only 0.030 lower than the apparent AUC. Bootstrap optimism correction yielded a mean optimism of 0.013 and an optimism-corrected AUC of 0.896, indicating no material overfitting. For individual genes, the apparent AUCs were 0.841 for CXCR2, 0.878 for S100A9, and 0.795 for LCN2, with LOOCV AUCs of 0.824, 0.862, and 0.765, respectively, and optimism values of 0.000, 0.002, and −0.001. Notably, in the IPAH dataset, LCN2 had the lowest apparent AUC among the three genes, suggesting that its perfect AUC (1.0) in the external validation cohort GSE130391 is more likely attributable to the extremely small validation sample size and batch effects than to true biological discrimination.

**TABLE 4:** Internal validation of the candidate classifier (n = 74).

Model	Apparent AUC	LOOCV AUC	Optimism	Optimism-corrected AUC
CXCR2	0.841	0.824	0.000	0.842
S100A9	0.878	0.862	0.002	0.876
LCN2	0.795	0.765	−0.001	0.795
Combined	0.909	0.879	0.013	0.896

LOOCV, leave-one-out cross-validation; optimism-corrected AUC, apparent AUC − optimism (bootstrap, 1,000 iterations); Combined = three-gene classifier (CXCR2 + S100A9 + LCN2).

### 3.7 Immune cell infiltration analysis

To investigate the characteristics of the IPAH immune microenvironment, we assessed the infiltration patterns of 28 immune cell types using ssGSEA. The results revealed significant alterations in IPAH patients. Specifically, compared with the control group, the Th17 cell infiltration score was significantly increased. In contrast, the infiltration scores of neutrophils, macrophages, MDSCs, activated dendritic cells, immature dendritic cells, CD56dim natural killer cells, and plasmacytoid dendritic cells were significantly decreased (Figure 8A). Spearman correlation analysis then evaluated the associations between the candidate biomarker genes and immune infiltration. The results showed that CXCR2 and S100A9 expression were both most strongly positively correlated with neutrophil infiltration, whereas LCN2 expression was most strongly positively correlated with activated dendritic cell infiltration (Figure 8B). It should be noted that these immune infiltration estimates were derived from transcriptomic deconvolution and have not been validated by orthogonal techniques such as flow cytometry or immunohistochemistry.

**FIGURE 8:**
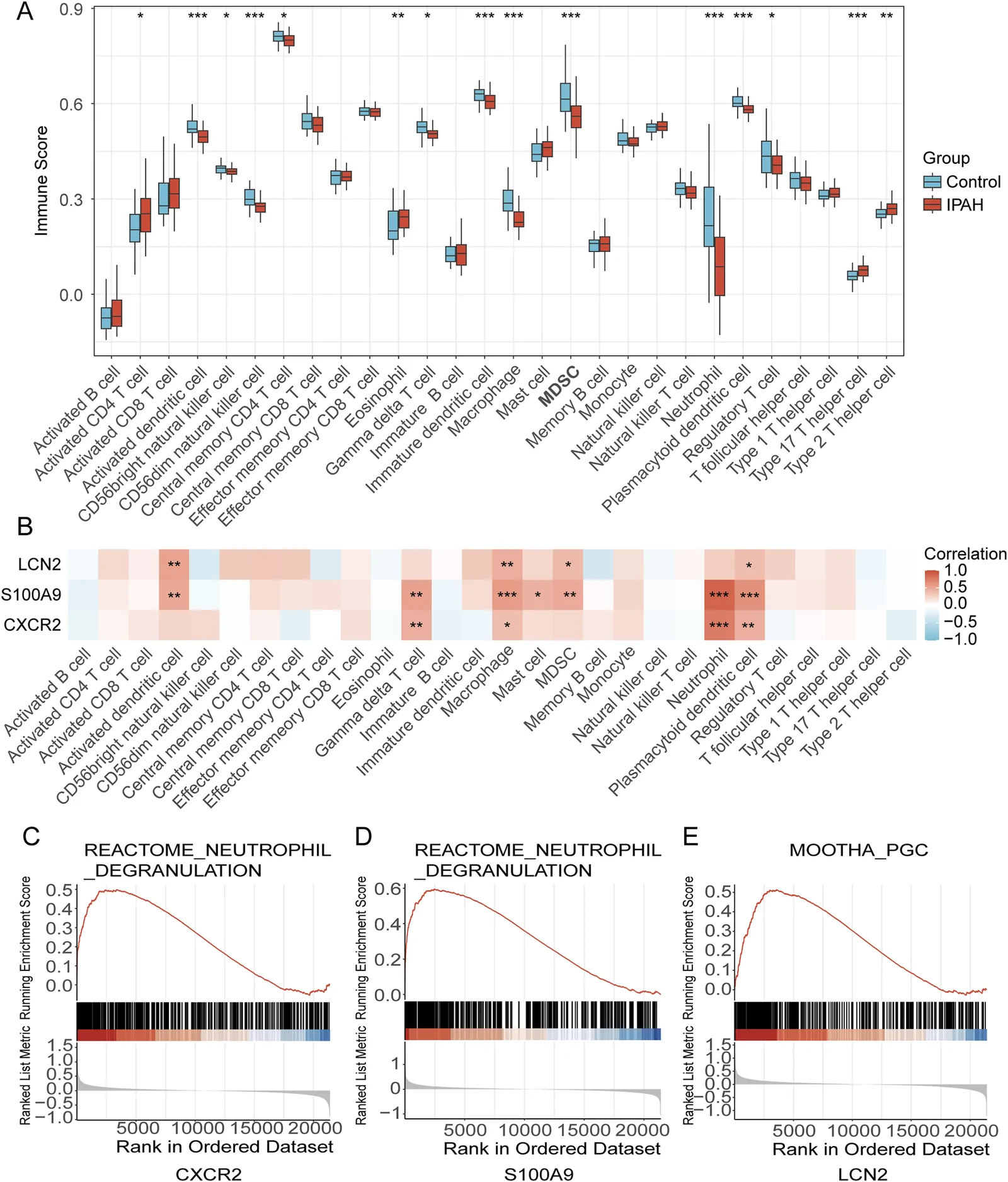
Immune infiltration and pathway enrichment analysis of IPAH candidate biomarker genes. **(A)** Immune infiltration scores of 28 cell types in IPAH versus control samples. **(B)** Correlation heatmap between the expression of the three candidate biomarker genes and immune cell infiltration scores. **(C–E)** GSEA plots for CXCR2, S100A9, and LCN2.

### 3.8 GSEA pathway enrichment analysis of candidate biomarker genes

In order to identify the biological pathways potentially regulated by the candidate biomarker genes, we used Gene Set Enrichment Analysis (GSEA) stratified by their expression profiles. The findings indicated that the expression signatures of both CXCR2 and S100A9 were markedly enriched in the neutrophil degranulation pathway (REACTOME_NEUTROPHIL_DEGRANULATION) (Figures 8C,D). The expression signature of LCN2 was significantly enriched in the MOOTHA_PGC pathway, a pathway related to cellular energy metabolism and mitochondrial function, suggesting its potential role in metabolic reprogramming (Figure 8E).

### 3.9 Molecular docking analysis of candidate drugs with candidate target proteins

Molecular docking was performed using the CB-Dock online server to evaluate the interactions between the identified candidate drugs and the candidate target proteins (CXCR2, S100A9, and LCN2). The binding energies are summarized in Table 5. For CXCR2, Navarixin Anhydrous showed the strongest binding affinity (−10.1 kcal/mol), followed by Danirixin (−9.6 kcal/mol), AZD-5122 (−9.3 kcal/mol), and Reparixin (−8.2 kcal/mol). For S100A9, Tasquinimod had a docking affinity of −7.2 kcal/mol, while Paquinimod showed −6.4 kcal/mol. For LCN2, Danirixin showed a binding energy of −7.7 kcal/mol, while Tasquinimod exhibited a binding energy of −7.3 kcal/mol. The binding mode of the CXCR2-Navarixin complex is shown in Figure 9. Docking results for all other ligand-protein pairs are provided in Figures S1-S3. These results suggest potentially favorable interactions between the candidate drugs and their respective candidate target proteins; however, docking predictions alone cannot establish therapeutic efficacy.

**TABLE 5:** Binding affinities of target proteins with their corresponding ligands.

Receptor	Entry id	Ligand	COMPOUND_CID	Affinity (kcal/mol)
CXCR2	P25025	Navarixin anhydrous	9865554	−10.1
		Danirixin	24780598	−9.6
		AZD-5122	44602493	−9.3
		Reparixin	9838712	−8.2
S100A9	P06702	Tasquinimod	54682876	−7.2
		Paquinimod	54684617	−6.4
LCN2	P80188	Danirixin	24780598	−7.7
		Tasquinimod	54682876	−7.3

**FIGURE 9:**
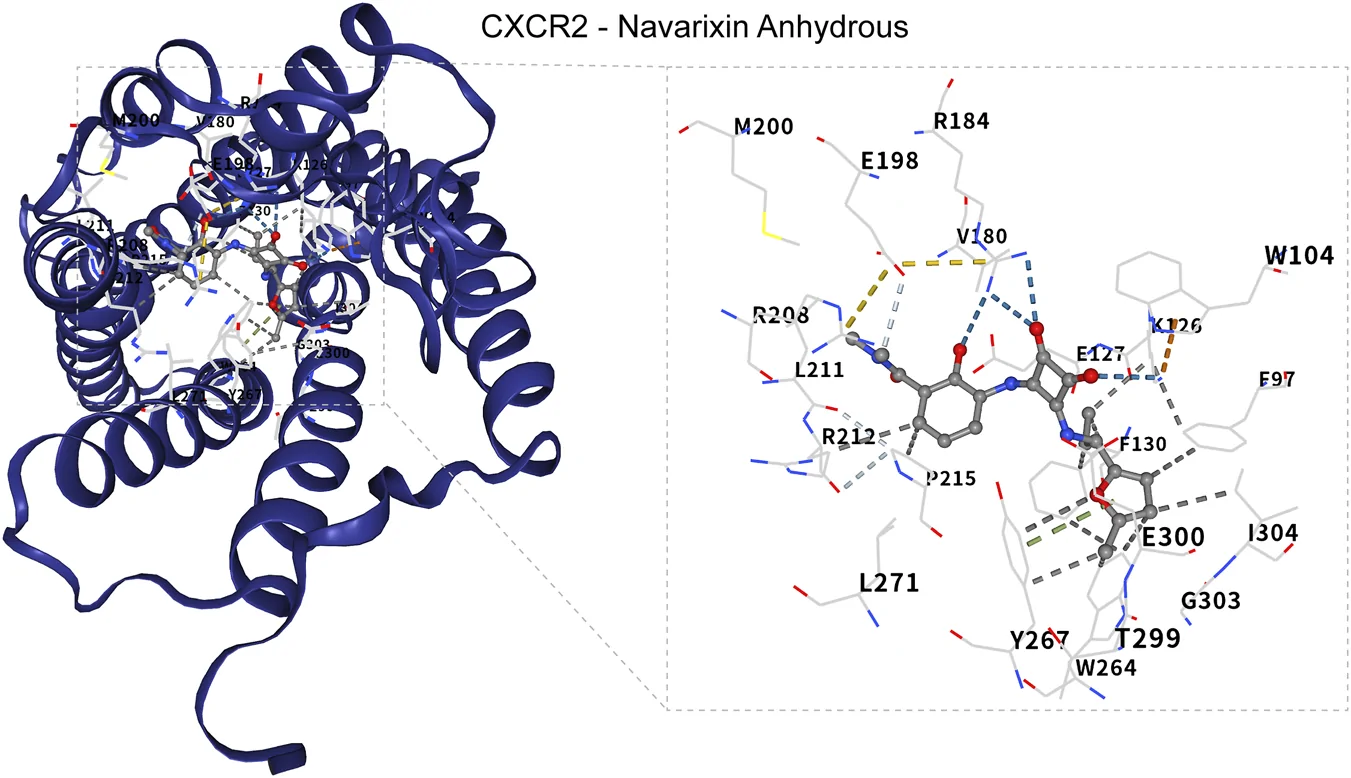
Molecular docking of the CXCR2-Navarixin complex.

### 3.10 Molecular dynamics simulations

In three independent 100 ns replicate MD simulations, we systematically assessed the conformational stability and binding mode consistency of the CXCR2-Navarixin complex. The protein backbone RMSD converged rapidly and remained stable at 0.3∼0.5 nm across all three replicates (Figure 10A). The radius of gyration (Rg) was maintained at 2.1 nm (Figure 10B), and the solvent-accessible surface area (SASA) remained steady at 170∼180 nm2 (Figure 10C), indicating the consistency in the overall structural compactness and solvent exposure. Residue-level RMSF revealed generally low receptor flexibility, with only small fluctuations in certain loop regions and no significant differences among the three replicates (Figure 10D). Hydrogen-bonding interactions between Navarixin and CXCR2 persisted throughout the simulations, averaging 2–3 hydrogen bonds (Figure 11A). The distances between the guanidino group of Arg184 (-NH2) and the O2 atom of Navarixin, as well as between the guanidino group of Arg208 (-NH2) and the O1 atom of Navarixin, remained consistently stable within the range of 0.4∼1.6 nm across all three replicates, further confirming the stable positioning of the ligand within the binding pocket (Figure 11B). Free energy landscape analysis demonstrated that all three replicates converged to a single low-energy conformational basin, with no appreciable conformational rearrangements or metastable transitions (Figure 12). Collectively, the three independent simulations exhibited high reproducibility, confirming that the CXCR2-Navarixin complex maintains a consistent binding mode on the 100 ns timescale.

**FIGURE 10:**
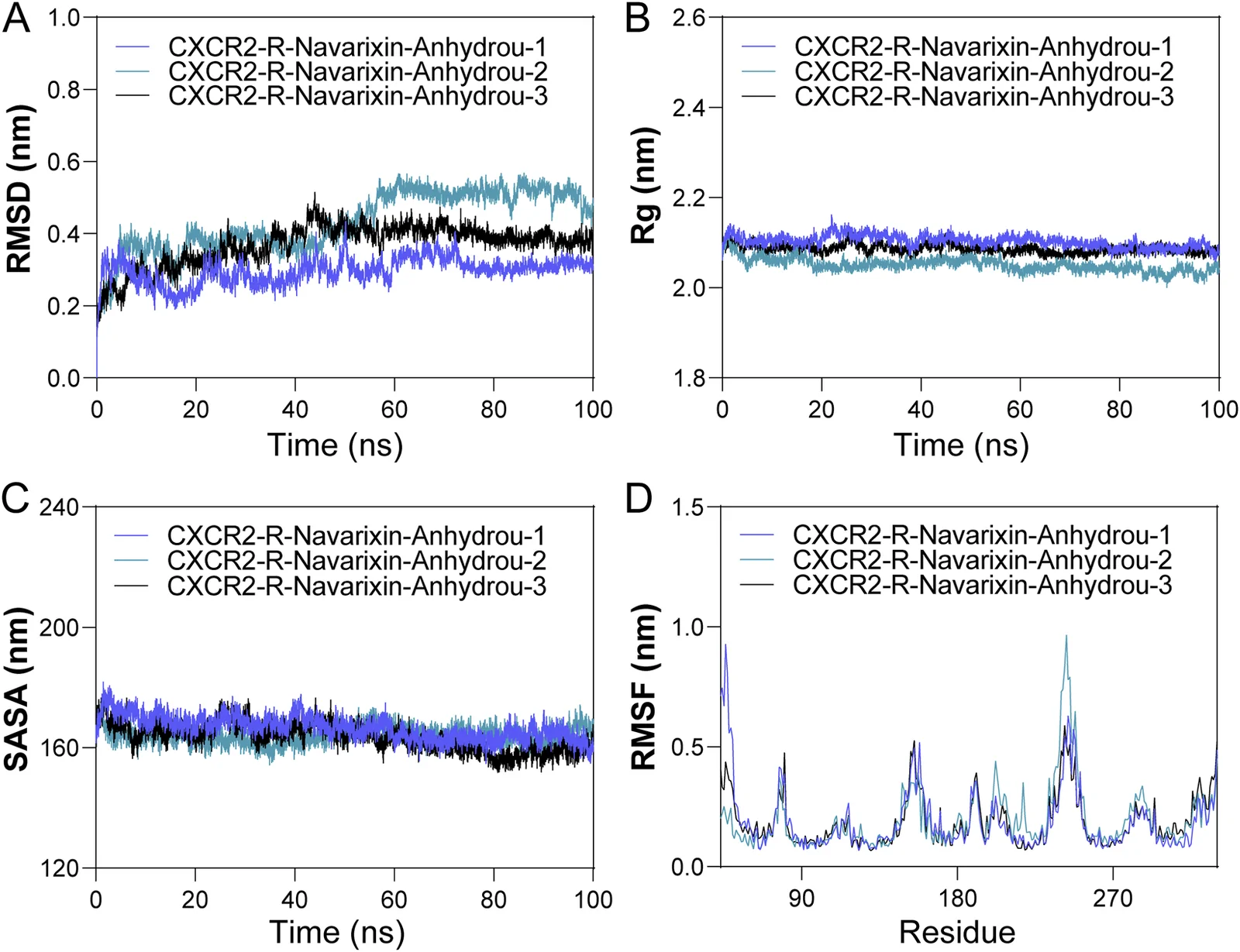
MD simulations of the CXCR2-Navarixin complex. **(A)** Time-dependent RMSD trajectories for the receptor backbone across three independent 100 ns replicates. **(B)** Rg reflecting structural compactness. **(C)** SASA assessing protein-solvent interactions. **(D)** RMSF of Cα atoms identifying flexible regions.

**FIGURE 11:**
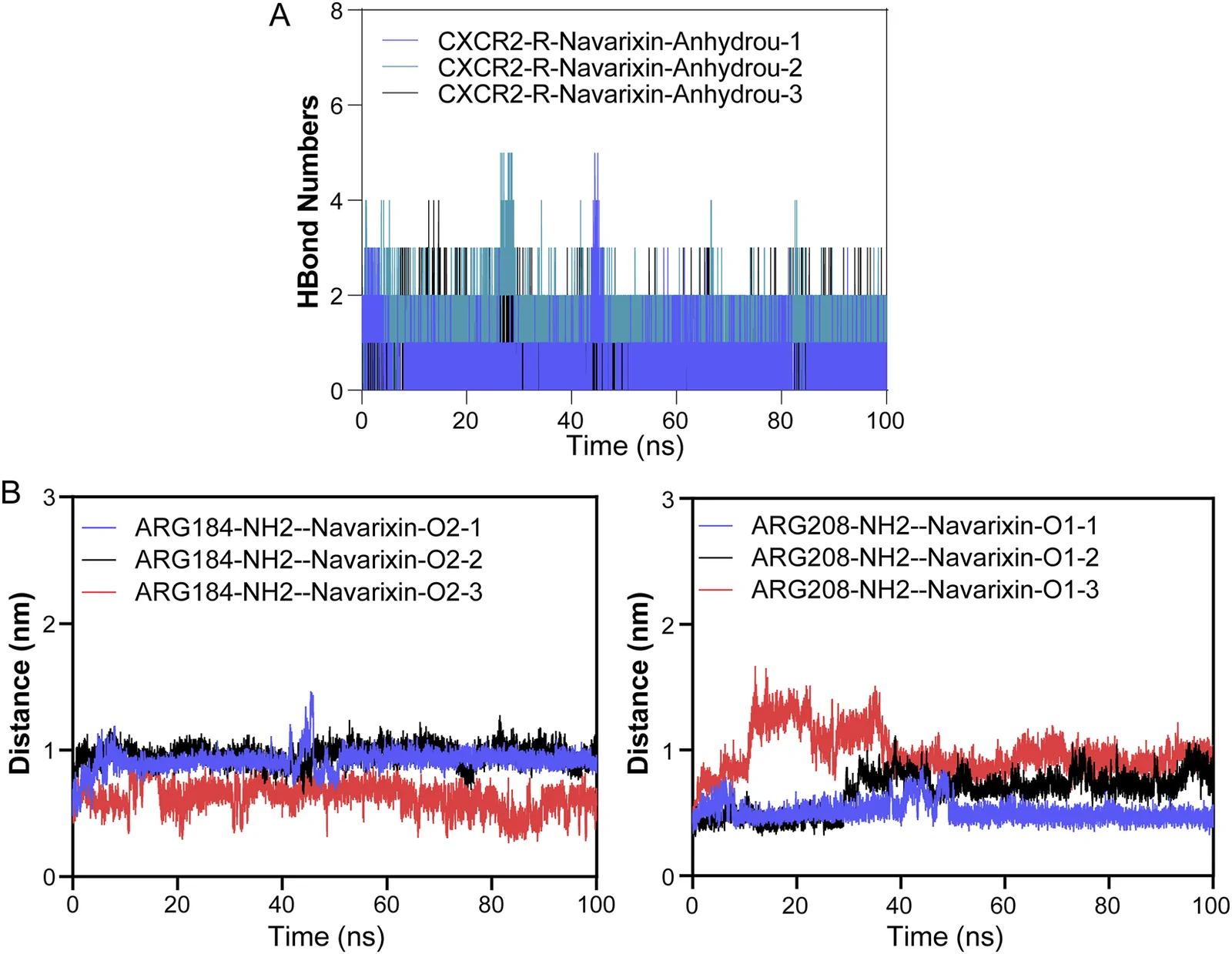
MD simulations of the CXCR2-Navarixin complex. **(A)** Time evolution of the number of hydrogen bonds between Navarixin and CXCR2. **(B)** Time-dependent distances between key binding pocket residues (Arg184-NH_2_ and Arg208-NH_2_) and specific oxygen atoms (O2 and O1) of Navarixin. Three different colors represent three independent replicate simulations.

**FIGURE 12:**
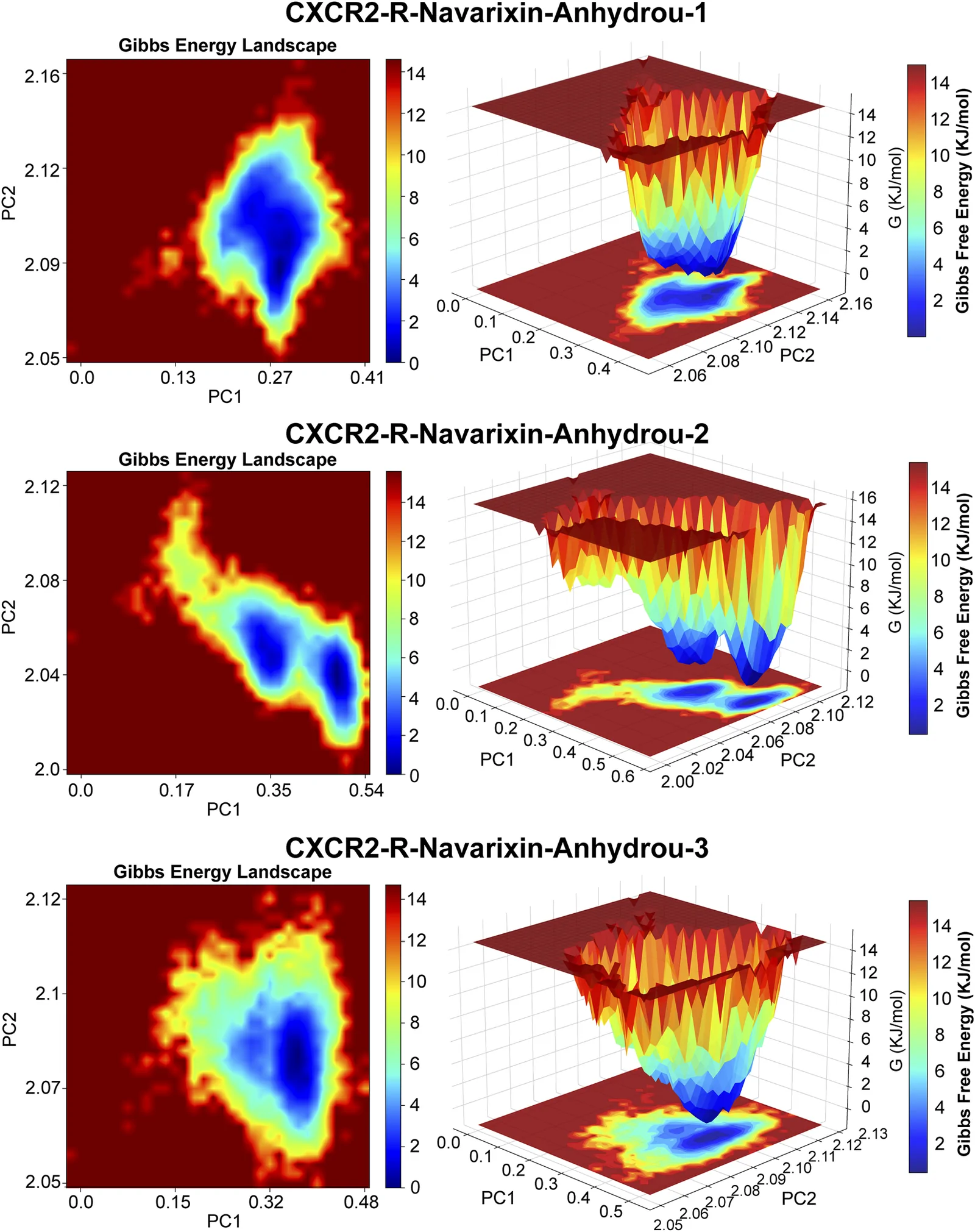
Gibbs free energy landscape of the CXCR2-Navarixin complex. The three panels (top to bottom) represent three independent replicate simulations. All replicates converged to a single low-energy conformational basin, indicating stable binding.

### 3.11 Validation of candidate biomarker gene expression by qRT-PCR

The mRNA expression levels of CXCR2, S100A9, and LCN2 were markedly reduced in peripheral blood samples obtained from individuals with IPAH (n = 8) when compared to those from healthy controls (n = 8) (Figures 13A–C, ***P < 0.001). These results aligned with the expression profiles seen in the training dataset (Figure 13D) and were additionally validated using the independent dataset GSE130391 (Figure 13E). The relative expression data for all three genes are provided in Supplementary Table S1. It should be noted that the qRT-PCR validation was performed in peripheral whole blood, whereas the transcriptomic discovery was based on lung or pulmonary vascular tissue; these findings therefore reflect circulating transcript levels and should not be directly extrapolated to the pulmonary vascular remodeling niche without tissue-level confirmation.

**FIGURE 13:**
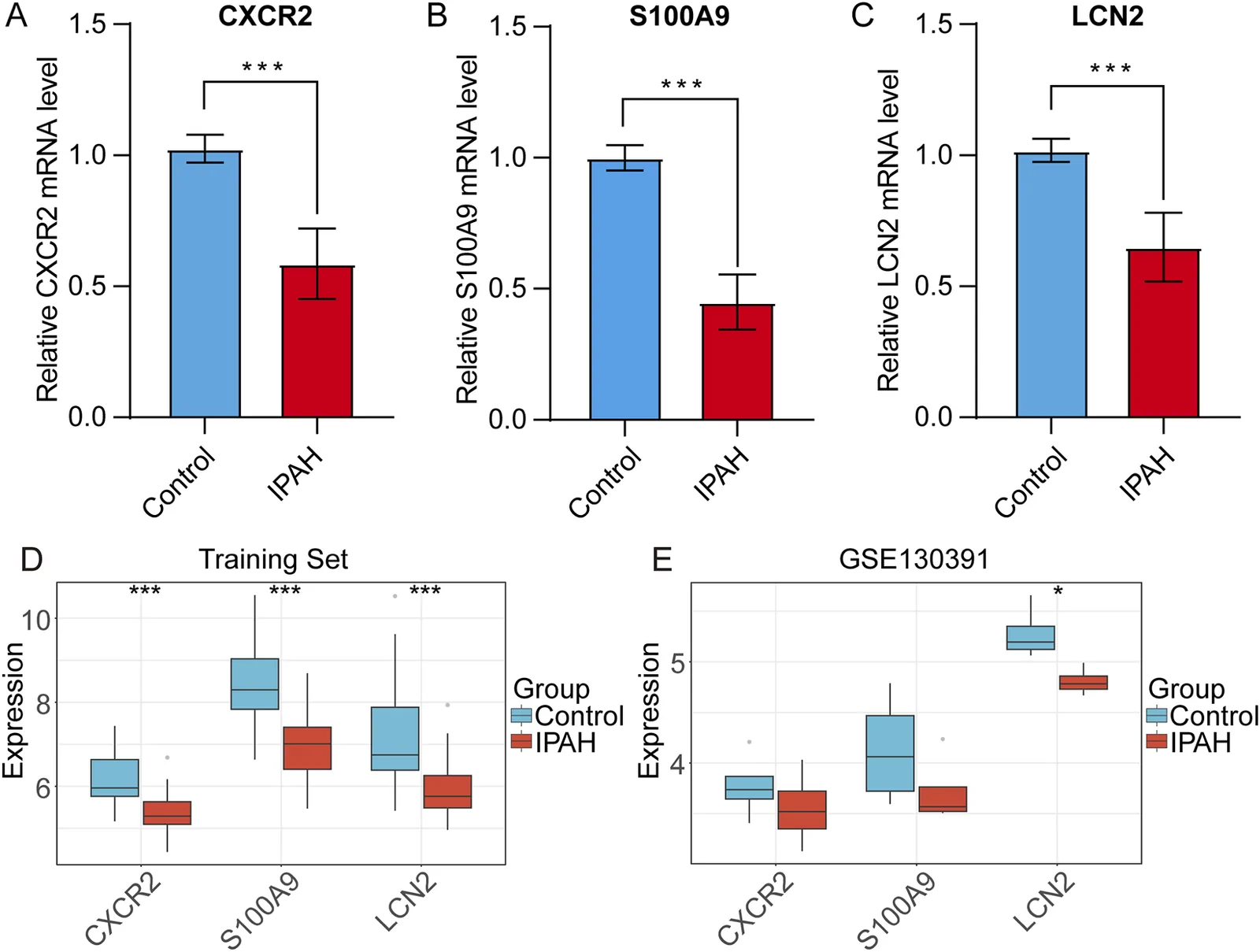
qRT-PCR validation and dataset expression analysis of candidate biomarker genes in IPAH. **(A–C)** Relative mRNA expression levels of CXCR2 **(A)**, S100A9 **(B)**, and LCN2 **(C)** in peripheral blood from the control group (n = 8) and the IPAH group (n = 8). ***P < 0.001 versus the control group. **(D)** Expression levels of the three candidate biomarker genes in the training dataset. **(E)** Expression levels of the three candidate biomarker genes in the independent validation dataset (GSE130391).

## 4 Discussion

IPAH is characterized by cancer-like features, including aberrant proliferation and apoptosis resistance, which contribute to its poor prognosis and persistently high mortality rates (Johnson et al., 2023; Chin et al., 2024). This highlights the urgent need to elucidate its molecular mechanisms. Given that IPAH exhibits cancer-like molecular characteristics and that MPM is a malignancy similarly involving HSP90 and matrix remodeling abnormalities, we hypothesized that the two conditions might share underlying molecular mechanisms. To test this hypothesis, we compared the transcriptomic profiles of IPAH and MPM using bioinformatics approaches. This analysis revealed, for the first time, shared DEGs and pathways between the two diseases, and identified CXCR2, S100A9, and LCN2 as candidate biomarker genes. Molecular docking and MD simulations were subsequently performed to assess the interaction potential of candidate drugs to these targets. CXCR2 and S100A9 are primarily involved in neutrophil chemotaxis and activation, whereas LCN2 is implicated in iron homeostasis and metabolic stress responses. These findings suggest that an interplay between neutrophil-associated immune dysregulation and metabolic reprogramming may contribute to the pathogenesis of IPAH.

Differential expression analysis identified six concordantly upregulated genes and eleven concordantly downregulated genes shared between IPAH and MPM (i.e., genes deregulated in the same direction in both diseases). The concordantly upregulated genes were enriched in pathways associated with cell cycle inhibition, such as negative regulation of G1/S transition. Given that a hallmark of IPAH is aberrant cell proliferation (Shen et al., 2024), this upregulation of inhibitory pathways presents an apparent paradox. This may reflect a compensatory or dysregulated negative feedback mechanism activated within the cell cycle under sustained proliferative stress. Such seemingly contradictory phenomena have been described in tumor biology, termed “oncogene-induced senescence” or stress-induced cell cycle arrest, characterized by compensatory upregulation of cell cycle inhibitors alongside enhanced proliferative signaling (Zhu et al., 2020). Since loss of cell cycle checkpoint control is a key feature of cancer proliferation (Liu et al., 2022c; Peng et al., 2025), these observations support the presence of cell cycle dysregulation in both conditions, consistent with the “cancer-like” hypothesis of IPAH.

In contrast, the concordantly downregulated genes were significantly enriched in innate immune pathways, including neutrophil chemotaxis, migration, and neutrophil extracellular trap formation. This downregulation pattern contrasts with the gene upregulation typically observed in acute inflammatory responses and differs markedly from the neutrophil activation reported in early-stage IPAH or experimental models (Fadón-Padilla et al., 2024; S et al., 2023; Ferrian et al., 2024). Notably, the IPAH samples analyzed in this study were derived from lung transplant recipients, representing end-stage disease, which may explain the discrepancies between our observations and those reported in earlier disease stages (Stacher et al., 2012). Consistent with this transcriptional profile, the immune infiltration analysis identified reduced levels of key innate immune cells, particularly neutrophils, in IPAH tissues. The concordance between transcriptional downregulation and reduced cellular infiltration suggests a distinct immune microenvironment in advanced IPAH. It is possible that this state reflects an adaptively regulated immune phenotype following early-stage overactivation, rather than a primary immune failure. However, because immune infiltration was inferred using transcriptomic deconvolution methods without functional immune assays or single-cell transcriptomic analyses, this interpretation should be considered preliminary. Recent single-cell transcriptomic studies of pulmonary hypertension have revealed substantial immune cell heterogeneity and dynamic inflammatory cell–vascular interactions that are not captured by bulk deconvolution approaches (Tang et al., 2024; Jiang et al., 2025), underscoring the need for orthogonal validation using flow cytometry, immunohistochemistry, or single-cell RNA sequencing to confirm these observations and distinguish between adaptive regulation and true immune exhaustion.

Through the integrated application of WGCNA, PPI network analysis, and machine learning screening approaches, we identified CXCR2, S100A9, and LCN2 as candidate biomarker genes shared between IPAH and MPM. These genes were selected computationally, and no functional or network perturbation experiments were performed; they should therefore be regarded as candidate biomarker or feature genes rather than established mechanistic hubs. CXCR2 and S100A9 are involved in regulating neutrophil recruitment and activation, and their downregulation is consistent with the reduced neutrophil infiltration observed in IPAH tissues, further supported by enrichment of the neutrophil degranulation pathway (Sprenkeler et al., 2022; Xie et al., 2023). LCN2 is known to participate in iron homeostasis and metabolic stress responses (Zhou et al., 2025). The downregulation of LCN2, together with enrichment of energy metabolism pathways, further implicates metabolic dysregulation in IPAH pathogenesis (Cuthbertson et al., 2023; Pokharel et al., 2023). The emerging literature on immune–metabolic crosstalk in pulmonary hypertension, including recent single-cell and spatial profiling studies, supports the view that immune and metabolic alterations are tightly coupled in this disease (Xu et al., 2025; Shi et al., 2026; Chen et al., 2026b). We propose that the coordinated downregulation of CXCR2, S100A9, and LCN2 may be associated with immune dysfunction and metabolic alterations in IPAH, and that this immuno-metabolic dysregulation may contribute to the “cancer-like” phenotype. This hypothesis requires direct functional testing.

These three candidate biomarker genes showed encouraging performance in a transcriptomic classification model. A model constructed based on CXCR2, S100A9, and LCN2 achieved AUC values of 0.909 and 0.875 in the training and external validation sets, respectively. Internal validation with LOOCV and bootstrap optimism correction confirmed that the model was not materially overfitted (LOOCV AUC 0.879; optimism-corrected AUC 0.896). Nevertheless, several caveats must be emphasized. First, this classifier was built from retrospective public datasets and has not been prospectively validated in a real-world clinical setting. It is best interpreted as an exploratory transcriptomic classification tool for hypothesis generation. Second, the external validation cohort comprised only four cases and four controls, and the perfect AUC of 1.0 observed for LCN2 is likely to be optimistic, potentially reflecting batch effects, overfitting, or the very small sample size rather than true biological discriminative power. Third, the available training and validation cohorts remain relatively limited in size. These findings were obtained from publicly available datasets and need confirmation in larger, independent, and multicentre populations before clinical translation can be considered.

In addition, another important caveat concerns the tissue origin of the datasets. The candidate biomarker genes were identified from lung or pulmonary vascular tissue transcriptomic datasets (GSE113439, GSE117261), whereas the qRT-PCR validation was performed on peripheral whole blood samples. The expression of these genes, particularly CXCR2 and S100A9, is cell-type dependent and dynamically regulated across different compartments (tissue-resident versus circulating immune cells). A downregulation in blood does not necessarily reflect, nor does it mechanistically inform, the transcriptional changes occurring in the pulmonary vascular remodeling niche. Recent single-cell analyses of peripheral blood in pulmonary hypertension have indeed identified distinct circulating immune phenotypes (Wu et al., 2023), and tissue-level confirmation of the candidate biomarker genes in pulmonary vascular cells is required before mechanistic conclusions can be drawn.

Molecular docking further evaluated candidate drugs targeting these candidate biomarker genes. The binding affinities of candidate drugs, including Navarixin, Danirixin, and Tasquinimod, to CXCR2, S100A9, and LCN2 ranged from −6.4 to −10.1 kcal/mol. Navarixin exhibited the strongest binding to CXCR2 (−10.1 kcal/mol). This binding mode was further examined by MD simulations: in three independent 100 ns replicate simulations, the CXCR2-Navarixin complex exhibited rapid convergence (RMSD 0.3∼0.5 nm), persistent hydrogen bonding (average 2∼3 bonds), and a single low-energy conformational basin. Consistent with this, Navarixin (SCH527123) is a potent oral CXCR2 antagonist that inhibits CXCR2-mediated chemotaxis and has been investigated for its immunomodulatory and anti-proliferative effects in preclinical and clinical studies (Armstrong et al., 2024). CXCR2 signaling is known to activate the MAPK/ERK pathway (Lazennec et al., 2024), which regulates G1/S cell cycle progression (Yoshizawa and Sako, 2025)—a process identified as dysregulated in our transcriptomic analysis of IPAH and consistent with prior reports of impaired cell cycle control in IPAH vasculature (Weiss et al., 2019). This convergence between computational predictions and transcriptomic findings suggests that targeting CXCR2 may be worthy of further investigation for modulating both immune and proliferative components of IPAH. However, docking and simulation results alone are insufficient to support therapeutic efficacy. Although the candidate biomarker genes were validated by qRT-PCR, the predicted drug-target interactions remain computational and should be interpreted cautiously until confirmed through functional experimental studies.

Several constraints of this study need to be recognized. First, the transcriptomic analyses relied on publicly available datasets with relatively limited sample sizes, which may constrain analytical sensitivity and diminish the generalizability of the observations. In particular, the MPM cohort was imbalanced (49 cases versus 9 controls). A ROTS sensitivity analysis at the matching FDR <0.05 threshold showed 100% overlap and direction concordance for all 1710 limma-identified DEGs, supporting the robustness of the DEG list, although validation in a more balanced cohort is still needed. Second, although the combination of WGCNA, various machine learning algorithms, molecular docking, and MD simulations established a comprehensive approach to pinpoint candidate biomarker genes and potential therapeutic agents, these findings remain correlative and predictive in nature and require experimental validation. Third, while the transcript levels of the candidate biomarker genes were validated in peripheral blood by qRT-PCR (n = 8 per group), this limited sample size may compromise the statistical robustness. Further confirmation in pulmonary vascular tissues, along with functional studies using lung tissues or primary pulmonary vascular cells, is needed to clarify the underlying mechanisms. Fourth, immune cell infiltration was inferred using transcriptomic deconvolution methods rather than direct experimental measurements, and validation using orthogonal techniques such as flow cytometry or single-cell RNA sequencing would strengthen the biological interpretation. Fifth, further validation in larger, independent, and multicentre cohorts is essential to confirm the classification performance of the candidate biomarkers and assess their clinical applicability.

## 5 Conclusion

This hypothesis-generating study identifies significant molecular commonalities at the transcriptomic level between IPAH and MPM through bioinformatics analysis. We identified CXCR2, S100A9, and LCN2 as shared candidate biomarker genes between the two diseases and constructed an IPAH classification model. The concordant downregulation of these genes is associated with reduced infiltration of immune cells, particularly neutrophils, and altered energy metabolism pathways in IPAH tissues. Molecular docking showed that candidate drugs including Navarixin, Danirixin, and Tasquinimod bind to these proteins, with the strongest affinity between Navarixin and CXCR2 (−10.1 kcal/mol). Three independent 100 ns replicate MD simulations consistently confirmed the stable binding of Navarixin to CXCR2 across all trajectories. While these computational findings suggest that CXCR2, S100A9, and LCN2 may form a network linking immune dysfunction and metabolic reprogramming that could contribute to the “cancer-like” phenotype in IPAH, these results remain correlative requiring confirmation through experimental studies and validation in larger independent cohorts before clinical implications can be drawn.

## Data Availability

The datasets presented in this study can be found in online repositories. The names of the repository/repositories and accession number(s) can be found in the article/Supplementary Material.

## Supplementary material

SUPPLEMENTARY FIGURE S1 Molecular docking of CXCR2 with additional candidate ligands. **(A)** CXCR2-Danirixin. **(B)** CXCR2-Reparixin. **(C)** CXCR2-AZD-5122.

SUPPLEMENTARY FIGURE S2 Molecular docking of S100A9 with candidate ligands. **(A)** S100A9-Tasquinimod. **(B)** S100A9-Paquinimod.

SUPPLEMENTARY FIGURE S3 Molecular docking of LCN2 with candidate ligands. **(A)** LCN2-Danirixin. **(B)** LCN2-Tasquinimod.

SUPPLEMENTARY TABLE S1 Raw qRT-PCR data for CXCR2, S100A9, and LCN2. The file contains three sheets: (1) CXCR2, (2) S100A9, and (3) LCN2. For each sheet, raw Ct values for the target gene and the internal control (ACTB) are provided for all 16 samples (8 controls and 8 IPAH patients). The raw data show that all samples yielded detectable Ct values within the expected range. These data were used to calculate relative gene expression via the 2^−ΔΔCT^ method, and the resulting expression values were used for statistical comparison between the IPAH and control groups.
