# Correction: Machine learning, molecular docking, and experimental validation identify shared molecular signatures between idiopathic pulmonary arterial hypertension and malignant pleural mesothelioma: a retrospective study

**DOI:** 10.3389/fgene.2026.2032350

**Published:** 2026-10-29

**Authors:** Neng Zhang, Chunyu Wang, Rui Wang, Xue Guo, Jing Yu, Jie Wang, Qin Luo, Yun Wu

**Affiliations:** 1 Department of General Medicine, The First Affiliated Hospital of Xinjiang Medical University, Urumqi, China; 2 Department of Emergency, Peking University People’s Hospital, Beijing, China; 3 College of Pharmacy, Xinjiang Medical University, Urumqi, China; 4 Department of Pharmacy, The First Affiliated Hospital of Xinjiang Medical University, Urumqi, China; 5 Department of Respiratory Neurology, The Second Affiliated Hospital of Xinjiang Medical University, Urumqi, China

**Keywords:** experimental validation, idiopathic pulmonary arterial hypertension, machine learning, malignant pleural mesothelioma, molecular docking

Affiliation 5 “Department of Respiratory Neurology, The Second Affiliated Hospital of Xinjiang Medical University, Urumqi, China” was omitted from the paper. This affiliation has now been added for author Qin Luo.

The original version of this article has been updated.

